# The Influence of Lead on Generation of Signalling Molecules and Accumulation of Flavonoids in Pea Seedlings in Response to Pea Aphid Infestation

**DOI:** 10.3390/molecules22091404

**Published:** 2017-08-24

**Authors:** Agnieszka Woźniak, Kinga Drzewiecka, Jacek Kęsy, Łukasz Marczak, Dorota Narożna, Marcin Grobela, Rafał Motała, Jan Bocianowski, Iwona Morkunas

**Affiliations:** 1 Department of Plant Physiology, Poznań University of Life Sciences, Wołyńska 35, 60-637 Poznań, Poland; agnieszkam.wozniak@gmail.com; 2 Department of Chemistry, Poznań University of Life Sciences, Wojska Polskiego 75, 60-625 Poznań, Poland; kinga.drzewiecka@gmail.com; 3 Chair of Plant Physiology and Biotechnology, Nicolaus Copernicus University, Gagarina 9, 87−100 Toruń, Poland; kesy@umk.pl; 4 Institute of Bioorganic Chemistry, Polish Academy of Sciences, Noskowskiego 12/14, 61-704 Poznań, Poland; lukasmar@ibch.poznan.pl; 5 Department of Biochemistry and Biotechnology, Poznań University of Life Sciences, Dojazd 11, 60-632 Poznań, Poland; dorna@o2.pl; 6 Department of Ecology and Environmental Protection, Laboratory of Environmental Analyses, the Institute of Plant Protection National Research Institute, Węgorka 20, 60−101 Poznań, Poland; grobela@iorpib.poznan.pl (M.G.); r.motala@iorpib.poznan.pl (R.M.); 7 Department of Mathematical and Statistical Methods, Poznań University of Life Sciences, Wojska Polskiego 28, 60-637 Poznań, Poland; jboc@up.poznan.pl

**Keywords:** lead, *Acyrthosiphon pisum*, signalling molecules, flavonoids, pisatin, flavonoid biosynthesis enzymes, defense responses, *Pisum sativum*

## Abstract

The aim of this study was to investigate the effect of an abiotic factor, i.e., lead at various concentrations (low causing a hormesis effect and causing high toxicity effects), on the generation of signalling molecules in pea (*Pisum sativum* L. cv. Cysterski) seedlings and then during infestation by the pea aphid (*Acyrthosiphon pisum* Harris). The second objective was to verify whether the presence of lead in pea seedling organs and induction of signalling pathways dependent on the concentration of this metal trigger defense responses to *A. pisum*. Therefore, the profile of flavonoids and expression levels of genes encoding enzymes of the flavonoid biosynthesis pathway (phenylalanine ammonialyase and chalcone synthase) were determined. A significant accumulation of total salicylic acid (TSA) and abscisic acid (ABA) was recorded in the roots and leaves of pea seedlings growing on lead-supplemented medium and next during infestation by aphids. Increased generation of these phytohormones strongly enhanced the biosynthesis of flavonoids, including a phytoalexin, pisatin. This research provides insights into the cross-talk between the abiotic (lead) and biotic factor (aphid infestation) on the level of the generation of signalling molecules and their role in the induction of flavonoid biosynthesis.

## 1. Introduction

Under natural conditions we may frequently observe the effect of many stress factors acting simultaneously or sequentially. Plants demonstrate a great ability to adapt their metabolism to rapid changes in the environment [1] and therefore they have developed a wide range of mechanisms to cope with abiotic and biotic stresses. It has been established that plant defenses against these stresses may imply common and/or complementary pathways of signal perception, signal transduction and metabolism [2,3]. Plants exposed to these stresses respond on multiple levels. To date molecular mechanisms involved in plant defenses against the above-mentioned stress factors were revealed independently and singly, thus further research is required to identify convergence points between abiotic and biotic stress signalling pathways. Fuijta et al. [4] reported that signalling molecules, transcription factors and kinases may be important common players that are involved in the crosstalk between stress signalling pathways. It has been suggested that phytohormone and reactive oxygen species (ROS)/ reactive nitrogen species (RNS) signalling pathways play key roles in the cross-talk between biotic and abiotic stress signalling. These cross-talk signalling pathways regulate metabolic processes in the context of plant defense. 

Since in the course of the coevolution of plants and biotic factors, including herbivores, undergo mutual adaptation, abiotic factors also significantly affect that process. Poschenrieder et al. [5] reported that during their coevolution with plants, pathogens and herbivores compete in an environment where efficient metal ion acquisition and ion homeostasis are essential for survival. Nevertheless, to date no studies have been conducted on plant-insect interactions involving the stress response signalling system in plants in relation to heavy metal concentration in the environment. Only plant signalling in response to herbivory, including aphids, has been well documented [6,7,8]. Invertebrates, especially insects, are good models to study heavy metal toxicity and can be bioindicators of environmental pollution. Insects, including aphids, play a definite role in the trophic chain and as food for other organisms they may constitute an important path for the bioaccumulation of heavy metals.

Studies in this work emphasise the important role of salicylic acid (SA) and abscisic acid (ABA) in defense responses associated with the accumulation of flavonoids in edible pea exposed to varying lead concentrations, i.e., at a low concentration inducing the metabolic status of the plants, potentially leading to the hormesis effect, and at a high concentration causing a toxic effect, as well as during infestation of *A. pisum*. This is the first report revealing the effect of lead as an abiotic factor and phytophages (*A. pisum*) as a biotic factor on the biosynthesis of pisatin, a phytoalexin characteristic of *Pisum sativum* L., which may serve a significant role in its defense strategy. Pisatin is believed to play a key role during abiotic and biotic stress responses. Jeandet et al. [9] reported that phytoalexins are biocidal compounds synthesised by and accumulated in plants as a response to biotic and abiotic stresses, which play important roles in their defense systems. Significantly enhanced production of phytoalexins was also observed in response to the elicitation of signalling molecules such as SA, methyl jasmonate and methyl-β-cyclodextrins in plants [10]. The induction of phytoalexin biosynthesis was demonstrated in many plant species in response to insects [11,12,13,14,15,16,17,18,19,20]. Dual-choice tests involving varied phytoalexin contents carried out by Hart [21] revealed that an isoflavonoid phytoalexin(s) had feeding-deterrent properties towards insects. Additionally, it has been revealed that several isoflavonoid phytoalexins, including coumestrol and genistein, deterred insect feeding [22,23]. The anti-nutritional effects of flavonoids on insects have also been confirmed by other research results [24,25,26]. Moreover, an isoflavone genistein and a flavone luteolin were shown to have an impact on the prolonged period of stylet probing, reduced salivation and passive ingestion of the pea aphid, *A. pisum* [27]. Simmonds [12,28] reported that flavonoids modulate the feeding and oviposition behaviour of insects. The aphicidal effect of flavonoids against aphids was manifested by mortality of nymphs and apterous adults [29]. It was suggested that flavonoids may be used as a bio-insecticide within the framework of integrated pest management (IPM) programmes. On the other hand, Diaz Napal and Palacios [30] demonstrated that flavonoids can also be phagostimulants when applied at a low concentration.

Moreover, the accumulation of phytoalexins was also demonstrated in plant responses to heavy metals [31,32,33,34,35,36,37]. The concentration of heavy metals, including lead, has been increasing in the environment as a result of progressive industrialisation. Ashraf et al. [38] reported that recent rates of soil contamination with various heavy metals leading to their introduction to agro-ecosystems and their transfer to human beings through the food chain are alarming and observed on a global scale. It has been documented that in terrestrial ecosystems soil is the primary source of heavy metal transfer to agricultural produce [39]. A proportion of these metals also enters plant systems from the external atmosphere surrounding the plants [40], thus affecting productivity and crop quality. Surface waters may also be contaminated with lead due to the use of nitrogen fertilisers containing this metal [41]. Pourrut et al. [42] reported that among heavy metals lead is the second most harmful pollutant, second only to arsenic, according to the new European REACH regulations. 

Edible pea, a crop object of our research, is used on a broad scale due to the high protein content in its seeds. Proper understanding of resistance mechanisms in crop plants is the foundation of integrated pest management. Additionally, insects playing a distinct role in the trophic chain and as food for other organisms may be an important element in the bioaccumulation of heavy metals.

The first objective was to investigate the effect of lead on the generation of signalling molecules such as phytohormones, e.g., SA and ABA, and next to determine how cross-interactions of both stress factors, i.e., lead and *A. pisum*, regulate the level of these signalling molecules and affect flavonoid biosynthesis. The second objective was to determine the level of flavonoids, especially a phytoalexin, pisatin, in response to the impact of the above-mentioned stressors. Flavonoids are a remarkable group of plant metabolites that are important elements of the defence system of legumes in interactions with biotic stress factors [14]. No other class of secondary products has been credited with so many and such diverse key functions in plants. Additionally, within the second research objective the level of expression was determined for genes encoding enzymes of the flavonoid biosynthesis pathway, i.e., phenylalanine ammonialyase (PAL), an enzyme initiating phenylpropanoid metabolism, and chalcone synthase (CHS), which catalyses the first committed step in the flavonoid biosynthetic pathway. It is known that flavonoids may be found either in a free state or conjugated as esters or glycosides. Biological activity in an interaction with biotic stressors was revealed by free flavonoid aglycones, released from glycosides with the use of glucosidases [43]. For this reason, in this study we analysed changes in the activity of β-glucosidase, an enzyme which hydrolyses flavonoid glucosides. In turn, PAL is an enzyme that catalyses a reaction converting L-phenylalanine to ammonia and *trans*-cinnamic acid. PAL is the first enzyme of the phenylpropanoid pathway, via which polyphenol compounds, such as flavonoids, are biosynthesised in plants. Additionally, this enzyme initiates one of the pathways of SA biosynthesis in plants [44]. Therefore, phenylalanine as a substrate is transformed by PAL to cinnamic acid, which then can be converted to *o*-coumaric acid, and in subsequent reactions to SA. Cinnamic acid may also be converted to benzoates, and then with the participation of the BA2H enzyme (benzoic acid-2-hydroxylase) to SA [45]. Moreover, the third objective was to determine the effect of lead at varying concentrations (i.e., at a low concentration inducing the metabolic status of the plants, potentially leading to the hormesis effect, and at a high concentration causing a toxic effect on the growth of pea seedlings. At the same time, we investigated lead content in roots and leaves of pea seedlings growing at varied lead concentrations in the medium and during cross-interactions of lead and infestation of a phytophage with the piercing-sucking mouthpart, i.e., *A. pisum*, as well as lead content in bodies of the insect *A. pisum*.

We assume in this study that at a low concentration of lead in the substrate this metal will be accumulated mainly in roots of pea seedlings, while at higher concentrations some of the accumulated lead will be transported to leaves. In the case of the application of these two different lead concentrations we may expect differences in the intensity of generation of signalling molecules such as SA and ABA, in the sequence/period of their generation, changes in the transduction of signals from roots to leaves and in triggering of defense responses, i.e., flavonoid accumulation, including pisatin. In view of the above we expect that at a low concentration lead deposited mainly in roots will induce synthesis of signalling molecules and the signal will be transmitted to leaves, which contributes to an enhanced stress defense potential of pea seedlings. In turn, in the case of the applied toxic concentration of lead in the substrate a certain pool of this metal will be transported from roots to leaves, thus physiological and biochemical changes in leaves of pea seedlings will firstly be a consequence of the direct effect of lead. 

## 2. Results

### 2.1. The Effect of Lead and A. pisum on SA Accumulation in Pea Seedlings

**Figure 1:**
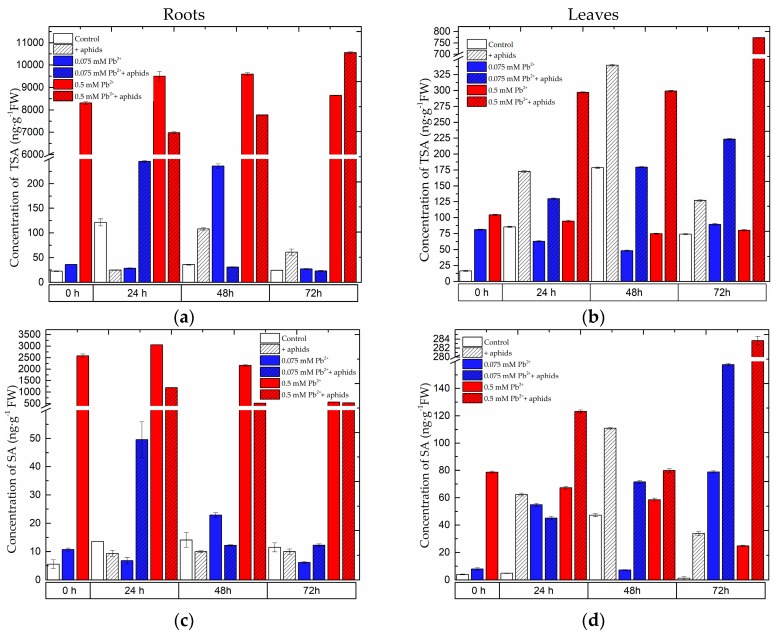

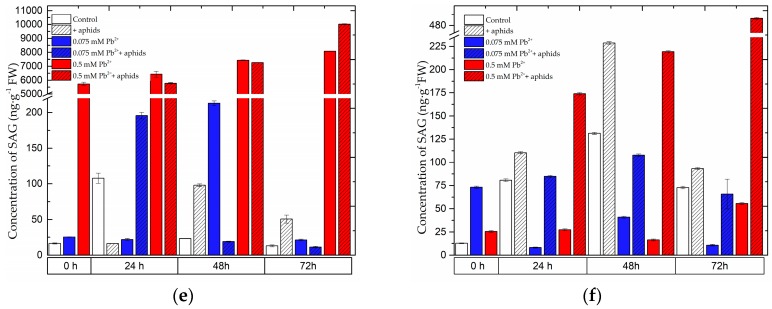
The effect of lead and *A. pisum* on accumulation of total salicylic acid (TSA) (**a,b**), salicylic acid (SA) (**c,d**) and salicylic acid glucoside (SAG) (**e,f**) in roots (**a,c,e**) and leaves (**b,d,f**) of pea seedlings. The data were obtained in three independent experiments and statistically analysed using ANOVA (*p*-values at α = 0.05). Hypotheses on the equality of means were verified by the two-sample *t*-test. To account for multiple testing, we used the Bonferroni correction (statistically significant differences are shown in Table S1).

Already at the beginning of the experiment, i.e., 4 days after the administration of lead, before aphid transfer to pea seedlings, a significant accumulation was recorded for total salicylic acid (TSA) (Figure 1a,b), i.e., the sum of free (SA) (Figure 1c,d) and glucoside-bound salicylic acid (SAG) (Figure 1e,f) in the roots and leaves of pea seedlings growing on the Hoagland medium with 0.075 and 0.5 mM Pb(NO_3_)_2_. Statistical analysis confirmed significance of differences in these results (Table S1a,b) in Supplementary Materials). Additionally, at 0 h of the experimental SA concentration in the roots of pea seedlings cultured at 0.075 and 0.5 mM Pb(NO_3_)_2_ was two and 500 times higher than in the control. In turn, at the application of 0.075 and 0.5 mM Pb(NO_3_)_2_ the level of SA in leaves of pea seedlings was 2- and 20-fold greater than in the control. The level of TSA in these organs of pea seedlings cultured at the high lead concentration (0.5 mM Pb(NO_3_)_2_ ) was significantly higher than in the organs of seedlings growing on the medium with 0.075 Pb(NO_3_)_2_ or in the control (pea seedlings cultured with no addition of lead and not colonised by pea aphids). The highest TSA level was recorded in 72-h roots (10,555.97 ng g^−1^ FW) and leaves (773 ng g^−1^ FW) of pea seedlings growing at the higher tested lead concentration of 0.5 mM Pb(NO_3_)_2_ and colonised by pea aphids *A. pisum*. The high accumulation of TSA was also found in the roots of pea seedlings growing on the Hoagland medium with 0.5 mM Pb(NO_3_)_2_ and not colonised by *A. pisum*. Moreover, pea aphid feeding significantly enhanced the accumulation of TSA in leaves, both in those of pea seedlings growing on the medium with a low lead level (0.075 mM Pb^2+^+aphids variant) and a high lead concentration (0.5 mM Pb^2+^+aphids variant), or without it (+aphids variant). In leaves of pea seedlings growing on the Hoagland medium with 0.075 mM Pb(NO_3_)_2_ and colonised by *A. pisum* the concentration of TSA increased versus infestation time, but it was significantly lower than in the case of leaves of seedlings cultured with the high lead concentration.

In 24-h roots of pea seedlings from the 0.075 mM Pb(NO_3_)_2_+aphids variant the level of free salicylic acid (SA) was observed to increase in comparison to the control, +aphids and +0.075 mM Pb^2+^ variants. In turn, in 48- and 72-h leaves the accumulation of SA in the 0.075 mM Pb^2+^+aphids variant was greater than in the control and 0.075 mM Pb(NO_3_)_2_ variants. Besides, the level of TSA in the roots was much higher than in the leaves. The A. pisum infestation alone caused a significant increase in the SA level, as the concentration of SA in these leaves was higher than in the control leaves.

### 2.2. The Effect of Lead and A. pisum on ABA Accumulation in Pea Seedlings

**Figure 2:**
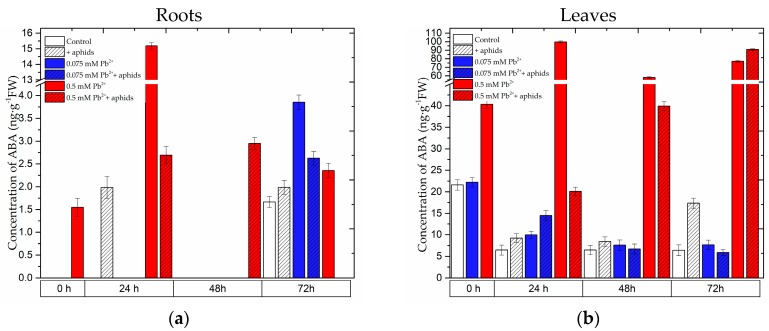
The effect of lead and *A. pisum* on accumulation of abscisic acid (ABA) in roots (**a**) and leaves (**b**) of pea seedlings. The data were obtained in three independent experiments and statistically analysed using ANOVA (*p*-values at α = 0.05). Hypotheses on the equality of means were verified by the two-sample *t*-test. To account for multiple testing, we used the Bonferroni correction (statistically significant differences are shown in Table S1).

The level of abscisic acid (ABA) was markedly higher in the leaves than in the roots (Figure 2a,b). At the application of the toxic lead concentration in the substrate (0.5 mM Pb(NO_3_)_2_) a very strong accumulation of ABA was observed already at the beginning of the experiment (0 h), i.e., after 4 days from the administration of lead at 0.5 mM Pb(NO_3_)_2_ . The greatest accumulation of ABA was recorded in 24-h roots (15 ng g^−1^ FW) and leaves (99.82 ng g^−1^ FW) of pea seedlings growing at the higher tested lead concentration of 0.5 mM Pb(NO_3_)_2_. Additionally, the high ABA level in these leaves (0.5 mM Pb^2+^ variant) was maintained at all time points of the experiment. An increase in ABA levels was also observed from 24 to 72 hpi in the leaves of pea seedlings growing on the medium with 0.5 mM Pb(NO_3_)_2_ and colonised by *A. pisum*, but only at 72 hpi it was higher than in the 0.5 mM Pb^2+^ variant and in the other variants. In turn, an increasing ABA level was also recorded in leaves of pea seedlings growing on the medium with the low lead level (0.075 mM Pb^2+^ variant) and infested by *A. pisum* (0.075 mM Pb^2+^+aphids variant), as the concentration of ABA in these leaves was 9.99 and 14.53 ng g^−1^ FW, respectively. In contrast, in the control it was 6.47 ng g^−1^ FW. Statistical analysis showed highly significant differences in these results (Table S1a,b). Also aphid feeding alone caused ABA accumulation in 72-h leaves. In addition, the highest ABA accumulation was recorded at 24 h of the experiment in the roots of the 0.5 mM Pb^2+^ variant. Up to 48 hpi ABA accumulation was also demonstrated in the roots of the 0.5 mM Pb^2+^+aphids variant. In turn, in the roots of pea seedlings growing on the medium with 0.075 mM Pb(NO_3_)_2_ an increase in ABA levels was recorded in comparison to the control, but only at 72 h of the experiment both in the 0.075 mM Pb^2+^ and 0.075 mM Pb^2+^+aphids variants. Additionally, ABA accumulation occurred also as a result of *A. pisum* feeding mainly at 24 hpi (+aphids variant).

### 2.3. The Effect of Lead and A. pisum on Accumulation of Flavonoids in Pea Seedlings

**Figure 3:**
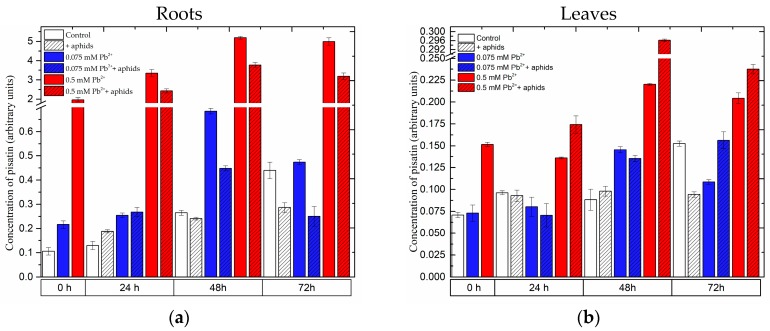
The effect of lead and *A. pisum* on accumulation of pisatin in roots (**a**) and leaves (**b**) of pea seedlings. The data were obtained in three independent experiments and statistically analysed using ANOVA (*p*-values at α = 0.05). Hypotheses on the equality of means were verified by the two-sample *t*-test. To account for multiple testing, we used the Bonferroni correction (statistically significant differences are shown in Table S1).

**Figure 4:**
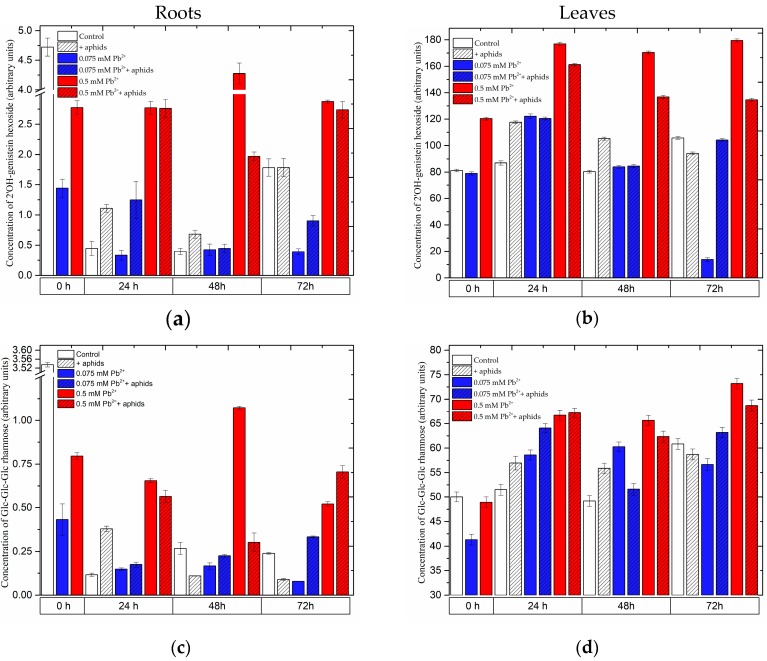

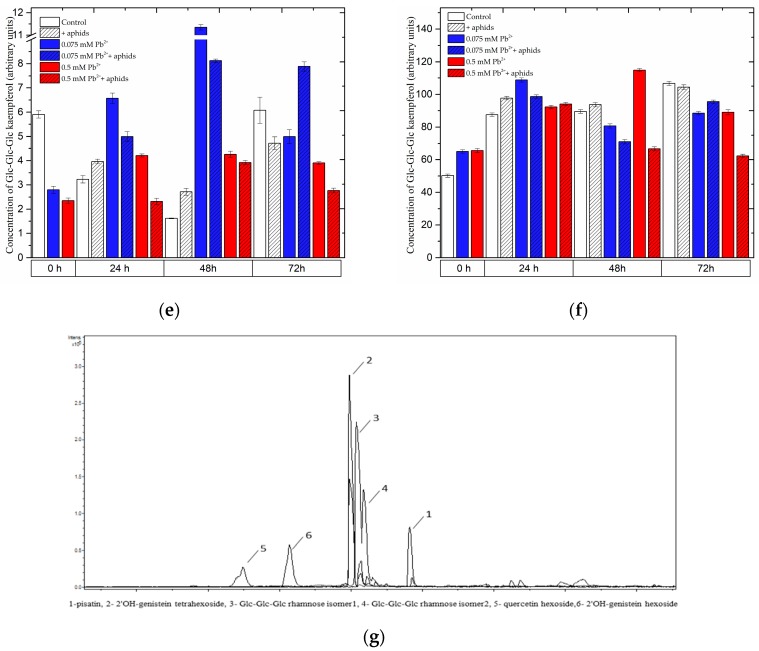
The effect of lead and *A. pisum* on the level of isoflavonoid and flavonoid glycosides 2’OH genistein hexoside (**a,b**), Glc-Glc-Glc rhamnose (**c,d**), Glc-Glc-Glc kaempferol (**e,f**), in roots (**a,c,e**) and leaves (**b,d,f**) of pea seedlings. An LC-MS extracted ion chromatogram showing profiles of phenolic componds found in leaves (**g**). Arbitrary unit means a relative unit of measurement to show the amount of substance (intensity), the reference measurement is dependent on individual measurement on MS (ion counts on MS detector). The data were obtained in three independent experiments and statistically analysed using ANOVA (*p*-values at α = 0.05). Hypotheses on the equality of means were verified by the two-sample *t*-test. To account for multiple testing, we used the Bonferroni correction (statistically significant differences are shown in Table S1).

The flavonoid profile in roots and leaves of pea seedlings revealed the presence of pisatin, 2’OH-genistein hexoside, Glc-Glc-Glc rhamnose, Glc-Glc-Glc kaempferol, Glc-Glc-Glc rhamnose isomer 1, Glc-Glc-Glc rhamnose isomer 2,2’OH-genistein tetrahexoside, quercetin hexoside, Glc-Glc-Glc kaempferol and Glc-Glc-Glc-Rha quercetin (Figure 3 and Figure 4, Figure S1).

#### 2.3.1. The Effect of Lead and *A. pisum* on Accumulation of Pisatin in Pea Seedlings 

It should be mentioned here that at the beginning of the experiment (i.e., 4 days after the administration of lead) the level of pisatin in the roots of the 0.5 mM Pb^2+^ variant was 19 times higher than in the control, while in the roots of the 0.075 mM Pb^2+^ variant its was two times higher than in the control (Figure 3a,b). Statistical analysis showed differences in these results to be highly significant (Table S1a,b). In turn, in leaves of the 0.5 mM Pb^2+^ variant at 0 h of the experiment the concentration of pisatin was 2-fold greater than in the other variants. Moreover, at all the time points of the experiment a significant pisatin accumulation was demonstrated in the organs of pea seedlings after lead administration at the high concentration. While a very strong pisatin accumulation was noted in the roots of the 0.5 mM Pb^2+^ and 0.5 mM Pb^2+^+aphids variants, in those of pea seedlings growing on the medium with 0.5 mM Pb(NO_3_)_2_ and not colonised by pea aphids (0.5 mM Pb^2+^ variant) the level of pisatin was higher than it was in the variant with 0.5 mM Pb(NO_3_)_2_ and pea aphids (0.5 mM Pb^2+^+aphids variant). In turn, in the roots of pea seedlings growing on the medium with 0.075 mM Pb(NO_3_)_2_ (0.075 mM Pb^2+^ and 0.075 mM Pb^2+^+aphids variants), the level of pisatin at 24 h and 48 h of the experiment was 2- and over 2.5-fold greater in relation to the control, respectively. Additionally, it should be mentioned here that pisatin level in the leaves of pea seedlings was lower than in roots. The highest pisatin accumulation was observed at 48 h of the experiment both in the roots and leaves of pea seedlings. However, in the leaves of the 0.5 mM Pb^2+^+aphids variant the level of this metabolite was highest. It should be stressed that the cross-talk of these two stressors, i.e., lead at 0.5 mM Pb(NO_3_)_2_ and pea aphid feeding, caused the strongest accumulation of pisatin at all time points after infestation. Additionally, from 48 hpi an increase was observed in pisatin levels in leaves of seedlings cultured at a low concentration of lead in the medium (0.075 mM Pb^2+^ variant). The cross-talk of lead at 0.075 mM Pb(NO_3_)_2_ and pea aphid infestation caused an accumulation of pisatin in leaves at 72 hpi, but this accumulation was significantly lower than in the leaves of the 0.5 mM Pb^2+^ and 0.5 mM Pb^2+^+aphids variants. 

#### 2.3.2. The Effect of Lead and *A. pisum* on the Level of Isoflavonoid and Flavonoid Glycosides in Pea Seedlings

Four days after the administration of lead at a high concentration (0 h of the experiment), before transferring aphids to pea seedlings, generally a significant accumulation of isoflavonoid and flavonoid glycosides was recorded (Figure 4, Figure S1 in Supplementary Materials). At this time point in the leaves of pea seedlings growing on the medium with 0.5 mM Pb(NO_3_)_2_, the levels of 2’OH-genistein hexoside, Glc-Glc-Glc rhamnose isomer 1, Glc-Glc-Glc rhamnose isomer 2,2’OH-genistein tetrahexoside, quercetin hexoside, Glc-Glc-Glc kaempferol and Glc-Glc-Glc-Rha quercetin were higher than in the other experimental variants (control and 0.075 mM Pb^2+^ variants). Statistical analysis showed highly significant differences in these results (Table S1a,b). In turn, an opposite trend was observed in the roots, i.e., a decrease in the content of the above-mentioned metabolites in relation to the control, with the exception of Glc-Glc-Glc rhamnose isomer 2, which was present only in the leaves (Figure 4, Figure S1). At subsequent time points both in the roots and leaves of pea seedlings, both non-infested and infested by *A. pisum*, growing on the medium with 0.5 mM Pb(NO_3_)_2_ (0.5 mM Pb^2+^ and 0.5 mM Pb^2+^+aphids variants), generally high accumulation was found for 2’OH-genistein hexoside, Glc-Glc-Glc rhamnose, Glc-Glc-Glc rhamnose isomer 1, Glc-Glc-Glc rhamnose isomer 2 (only in the leaves) and 2’OH-genistein tetrahexoside. However, concentrations of theses metabolites in tissues affected by both factors, i.e., abiotic (lead) and biotic (aphid) (0.5 mM Pb^2+^+aphids variant), were lower than in those affected by lead alone (0.5 mM Pb^2+^ variant). At 24 h of the experiment, during the influence of only lead applied at the low concentration (0.075 mM Pb^2+^ variant), as well as cross-talk between lead and *A. pisum* (0.075 mM Pb^2+^+aphids variant) and only *A. pisum* infestation (+aphids), levels of such metabolites as 2’OH-genistein hexoside, 2’OH-genistein tetrahexoside, Glc-Glc-Glc rhamnose, Glc-Glc-Glc rhamnose isomer 1, Glc-Glc-Glc rhamnose isomer 2, quercetin hexoside, Glc-Glc-Glc kaempferol and Glc-Glc-Glc-Rha quercetin were observed to increase in leaves of pea seedlings. Besides, generally at 48 hpi in leaves of pea seedlings infested by *A. pisum* and growing on the medium with 0.075 mM Pb(NO_3_)_2_ the levels of 2’OH-genistein tetrahexoside, Glc-Glc-Glc rhamnose, Glc-Glc-Glc rhamnose isomer 1, Glc-Glc-Glc rhamnose isomer 2, Glc-Glc-Glc kaempferol and Glc-Glc-Glc-Rha quercetin decreased in relation to those in leaves of the 0.075 mM Pb^2+^ variant. Moreover, at 72 h of the experiment, i.e., 7 days after lead application, levels of these metabolites in the leaves and roots of pea seedlings cultured on the medium with 0.075 mM Pb(NO_3_)_2_ (0.075 mM Pb^2+^ variant) were markedly reduced in relation to the control.

#### 2.3.3. Quantitative Analysis of Metabolites in the Roots and Leaves of Pea Seedlings

**Figure 5:**
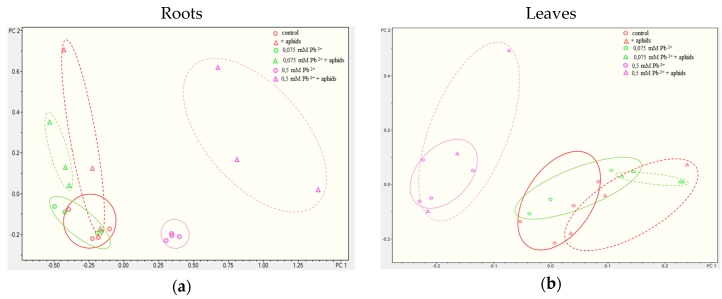

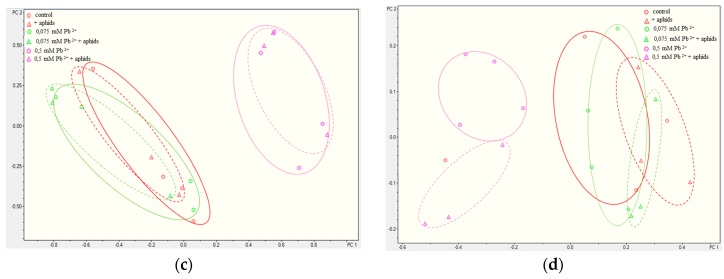
Quantitative analysis of metabolites in roots (**a,c**) and leaves (**b,d**) of pea seedlings. The Principal Components Analysis (PCA) of the positive ion (**a,b**) and negative ion (**c,d**) MS shows distinct and close clustered groups of these ions for experimental variants, i.e., control, +aphids, 0.075 mM Pb^2+^, 0.075 mM Pb^2+^+aphids, 0.5 mM Pb^2+^ and 0.5 mM Pb^2+^+aphids

Comparative metabolomic analyses were performed between each group of samples (variants) both for roots and for leaves (Figure 5). PCA differentiated all the analysed experimental groups, whereas the group of samples from pea seedlings treated with the higher lead concentration was very clearly separated in the case of roots, while the additional treatment with aphids also gave a distinctly separated group. Such good separation was not the case for pea seedling leaves, although also in this case samples obtained from pea seedling leaves treated with the higher lead concentration are easily distinguished from the others. In contrast, the effect of aphid presence on the metabolome was not as evident as in the case of roots. In turn, in leaves significant changes were observed between the metabolome of such groups as 0.5 mM Pb^2+^, 0.5 mM Pb^2+^+aphids and 0.075 mM Pb^2+^, 0.075 mM Pb^2+^+aphids, +aphids. Moreover, it is interesting that quantitative analyses of metabolites for roots demonstrated that the control group, the group growing in the medium with the low lead concentration (0.075 mM Pb^2+^ variant ) and the group only infested by pea aphids ( +aphids variant) were clustered close to each other. Only the 0.5 mM Pb^2+^+aphids variant was located separately, although it was still near them.

Unfortunately, it was impossible to identify all the differentiating components of the sample due to the difficulty in interpreting the MS2 spectra, although spectral analysis showed that partially differentiation of the samples corresponded to flavonoid derivatives. For this reason, the MS2 spectra of flavonoids were analysed both for positive and negative ions and the quantity of identified compounds was measured using the targeted analysis approach by determining the areas of the corresponding chromatographic peaks.

### 2.4. The Effect of Lead and A. pisum on Expression Levels of Phenylalanine Ammonialyase and Chalcone Synthase Genes in Pea Seedlings

**Figure 6:**
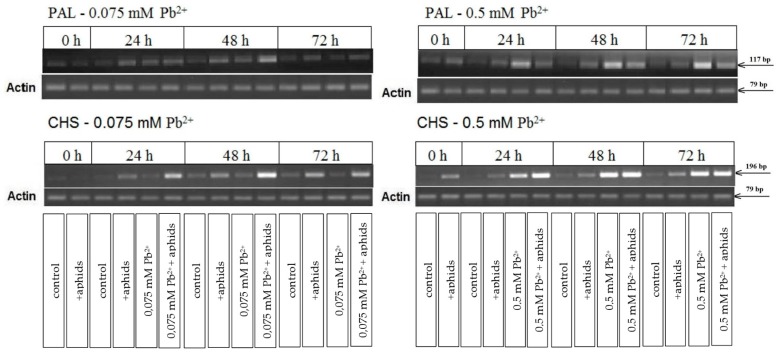
The effect of lead and *A. pisum* on expression levels of phenylalanine ammonialyase (PAL) and chalcone synthase (CHS) genes in leaves of pea seedlings. The data were obtained in three independent experiments and statistically analysed using ANOVA (*p*-values at α = 0.05). Hypotheses on the equality of means were verified by the two-sample *t*-test. To account for multiple testing, we used the Bonferroni correction (statistically significant differences are shown in Table S1).

Semi-quantitative RT-PCR analysis revealed that pea aphid feeding alone (+aphids), lead administration at the high and low concentrations in the medium (0.5 mM Pb^2+^ and 0.075 mM Pb^2+^) as well as the cross-talk of lead and *A. pisum* infestation (0.5 mM Pb^2+^+aphids and 0.075 mM Pb^2+^+ aphids) upregulated mRNA levels for PAL and CHS in relation to the control (Figure 6). However, during exposure to lead at the high concentration and *A. pisum* (0.5 mM Pb^2+^+aphids variant), the expression level of genes encoding PAL in the 0.5 mM Pb^2+^+aphids leaves was lower than in the 0.5 mM Pb^2+^ leaves, while it was generally higher than in leaves infested by aphids only (+aphids variant). Besides, the low concentration of lead (0.075 Pb(NO_3_)_2_) caused also upregulation of mRNA levels of the PAL and CHS genes. At the application of the low lead concentration the highest relative mRNA level for PAL was observed at 48 hpi in the leaves of seedlings growing on the medium with 0.075 Pb(NO_3_)_2_ and colonised by pea aphids (0.075 mM Pb^2+^+aphids variant). However, these stress factors had a much stronger effect on the upregulation of CHS rather than PAL. The very high upregulation of mRNA for the CHS genes was observed as a result of the impact of lead or the cross-talk of lead and *A. pisum*, especially at the toxic concentration of lead. Also, the low lead level and *A. pisum* infestation (0.075 mM Pb^2+^+aphids variant) raised the mRNA level for CHS, but it was much less markedly than in the case of lead or the cross-talk of lead at the toxic concentration and *A. pisum*.

### 2.5. The Effect of Lead and A. pisum on β-Glucosidase Activity in Pea Seedlings

**Figure 7:**
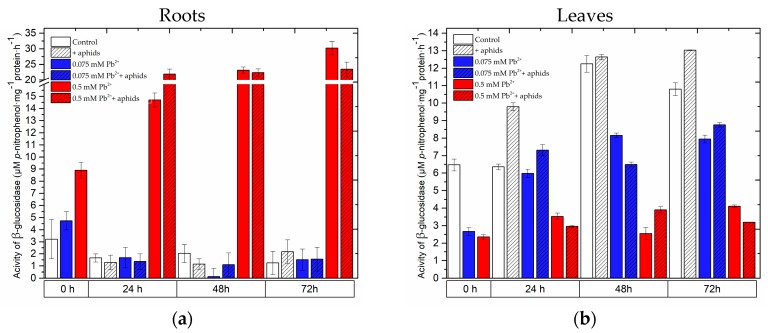
The effect of lead and *A. pisum* on β-glucosidase activity in roots (**a**) and leaves (**b**) of pea seedlings. The data were obtained in three independent experiments and statistically analysed using ANOVA (*p*-values at α = 0.05). Hypotheses on the equality of means were verified by the two-sample *t*-test. To account for multiple testing, we used the Bonferroni correction (statistically significant differences are shown in Table S1).

In the roots of pea seedlings growing on the Hoagland medium supplemented with lead, already after 4 days from the administration of lead an increase in β-glucosidase activity was observed, being higher at the high lead concentration in comparison to that at the low lead concentration (Figure 7, Table S1a,b). A very high stimulation of β-glucosidase activity was recorded in roots of pea seedlings of the 0.5 mM Pb^2+^ and 0.5 mM Pb^2+^+aphids variants. At 24 hpi during the cross-talk of lead (0.5 mM Pb(NO_3_)_2_) and *A. pisum*, the activity of this enzyme in roots was highest among the experimental variants tested and this high level was maintained at subsequent time points after infestation. Moreover, it is of interest that at 72 hpi this activity dropped in roots of the 0.5 mM Pb^2+^+aphids variant in relation to the activity of β-glucosidase in roots of the 0.5 mM Pb^2+^ variant. A similar trend was observed in leaves at 72 hpi. In turn, the activity of the enzyme in leaves of seedlings cultured on the medium with lead was lower than in the control at 0 h of the experiment. At 48 and 72h under the influence of lead alone applied at the low concentration and the cross-talk of lead and *A. pisum* (0.075 mM Pb^2+^ and 0.075 mM Pb^2+^+aphids variants), a reduction in β-glucosidase activity was observed in relation to the control. Additionally, aphid infestation alone (+aphids variant) stimulated an increase in β-glucosidase activity, as at 24 and 72 hpi the activity of this enzyme was higher than in the control. It should be added that the lowest activity of the enzyme was recorded in the leaves of pea seedlings growing on the medium with lead applied at the high concentration (0.5 mM Pb^2+^ variant) and in leaves of the 0.5 mM Pb^2+^+aphids variant.

### 2.6. The Effect of Lead and A. pisum on Activity of Phenylalanine Ammonialyase in Pea Seedlings

**Figure 8:**
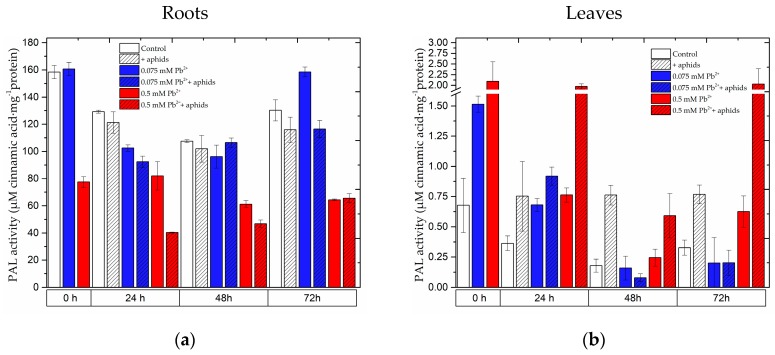
The effect of lead and *A. pisum* on activity of phenylalanine ammonialyase in roots (**a**) and leaves (**b**) of pea seedlings. The data were obtained in three independent experiments and statistically analysed using ANOVA (*p*-values at α = 0.05). Hypotheses on the equality of means were verified by the two-sample *t*-test. To account for multiple testing, we used the Bonferroni correction (statistically significant differences are shown in Table S1).

The activity of phenylalanine ammonialyase (PAL) differed in the roots and the leaves of pea seedlings (Figure 8). At all time points of the experiments a decrease in PAL activity was observed in the roots of pea seedlings exposed to stress factors in relation to the control, with the exception of the activity of this enzyme in roots exposed to lead at the low concentration at 72 h (0.075 mM Pb^2+^ variant). It should be noted that the strong reduction in enzyme activity was recorded in roots of pea seedlings exposed to lead alone at the higher concentration (0.5 mM Pb^2+^ variant) and during the cross-talk of lead and the pea aphid (0.5 mM Pb^2+^+aphids variant). In turn, during the impact of these stress factors, i.e., aphid infestation (+aphids), lead at the low concentration (0.075 mM Pb^2+^) and the cross-talk of lead and aphids (0.075 mM Pb^2+^+aphids), the reduction in PAL activity in roots of seedlings exposed to these stress factors was not marked when compared to the control. Moreover, in leaves of seedlings growing for 4 days on the medium with lead (0 h of the experiment), PAL activity increased both at the low and at the high lead concentration, although it was higher in leaves growing on the medium with 0.5 mM Pb(NO_3_)_2_ than in leaves growing on the medium with 0.075 mM Pb(NO_3_)_2_. At subsequent time points a higher PAL activity was detected in leaves infested by aphids (+aphids variant) and in leaves of seedlings growing on the medium supplemented with lead at the high concentration (0.5 mM Pb^2+^ variant) as well as during the cross-talk of lead and A. pisum (0.5 mM Pb^2+^+aphids variant) in relation to the control. A very high PAL activity was observed at 24 and 72 h of the experiment in the seedlings growing on the medium with 0.5 mM Pb(NO_3_)_2_ and infested by pea aphids (0.5 mM Pb^2+^+aphids variant). For lead application at the low concentration, at 48 h and 72 h of the experiment in leaves of pea seedlings PAL activity was found to decrease in relation to the control. Statistical analysis showed significant differences in these results (Table S1a,b). Additionally, it should be mentioned that after 4 days from the administration of the high lead concentration (0 h of the experiment), a 2-fold decrease in enzyme activity was recorded.

### 2.7. The Effect of Lead and A. pisum on the Growth of Pea Seedlings

**Figure 9:**
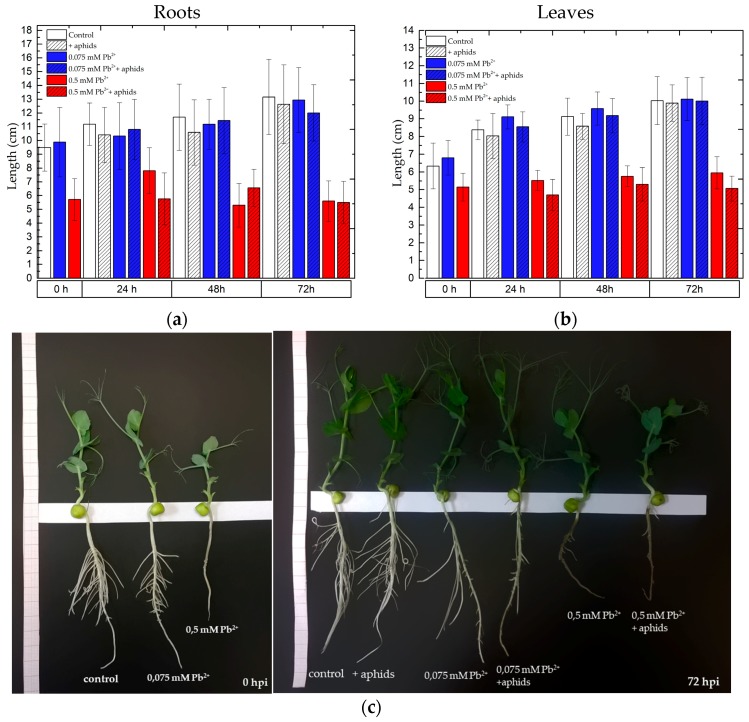
The effect of lead and *A. pisum* on growth of roots (**a**) and shoots (**b**) of pea seedlings. Pea seedlings after 4 days from the administration of lead and prior to transferring aphids onto pea seedlings (at 0 h) and 72 h after pea aphid infestation; (**c**). The data were obtained in ten independent experiments and statistically analysed using ANOVA (*p*-values at α = 0.05). Hypotheses on the equality of means were verified by the two-sample *t*-test. To account for multiple testing, we used the Bonferroni correction (statistically significant differences are shown in Table S1).

The administration of lead to the medium caused symptoms of phytotoxicity, which were varied and depended on the concentration of lead in the medium. Symptoms of phytotoxicity were visible the earliest on the roots due to their direct contact with lead ions (results not shown). Calculations of the Index of Tolerance (IT) showed that among the tested lead concentrations (0.05 and 0.075 mM Pb(NO_3_)_2_) inhibition of root elongation in relation to the control was found at the concentration of 0.5 mM Pb(NO_3_)_2_. Additionally, feeding of *A. pisum* on pea seedlings cultured with an addition of lead at the above-mentioned concentrations only slightly reduced elongation of pea seedlings (Figure 9).

We need to stress here that it was the lower of the tested lead concentration, i.e., 0.075 mM Pb(NO_3_)_2_. The effect of lead alone at the above concentration, as well as the simultaneous effect of lead (0.075 mM) and *A. pisum* generally did not inhibit the increment in length of pea seedlings, as the values of these growth indexes were only slightly higher than in the control. Stimulation of shoot and root length of pea seedlings by the low lead concentration (0.075 mM Pb(NO_3_)_2_) was visible at the beginning of the experiment (after 4 days from the administration of lead before transferring the aphids to pea seedlings) and at the subsequent time points, i.e., 24 h and 48 h for shoots. In contrast, a significant inhibition of root and shoot growth of pea seedlings was found after 4 days from the administration of lead at the high concentration. The roots in the 0.5 mM Pb^2+^ variant were two times shorter than those of the control and the roots of 0.075 mM Pb^2+^ variants. At subsequent time points of the experiment (24, 48 and 72 hpi) limitation of root elongation growth was also found in the 0.5 mM Pb^2+^ and 0.5 mM Pb^2+^+aphids variants. Therefore, the applied toxic concentration (0.5 mM Pb(NO_3_)_2_) caused the strong inhibition of growth in pea seedlings, while the applied low concentration (0.075 mM Pb(NO_3_)_2_) caused stimulation of growth in pea seedling shoots up to 48 h, with these differences being statistically significant (Table S1a,b).

### 2.8. The Content of Lead in the Roots and Leaves of Pea Seedlings and in Bodies of Pea Aphid 

**Figure 10:**
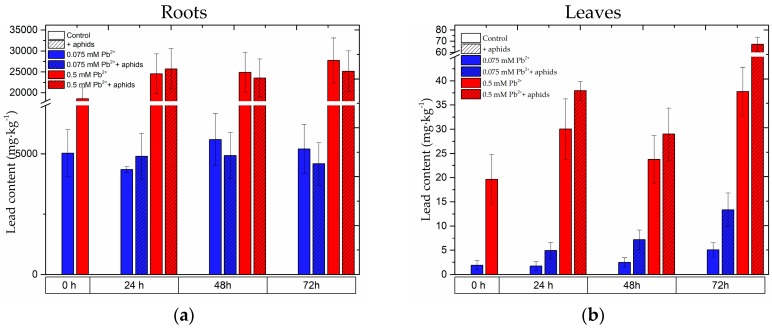

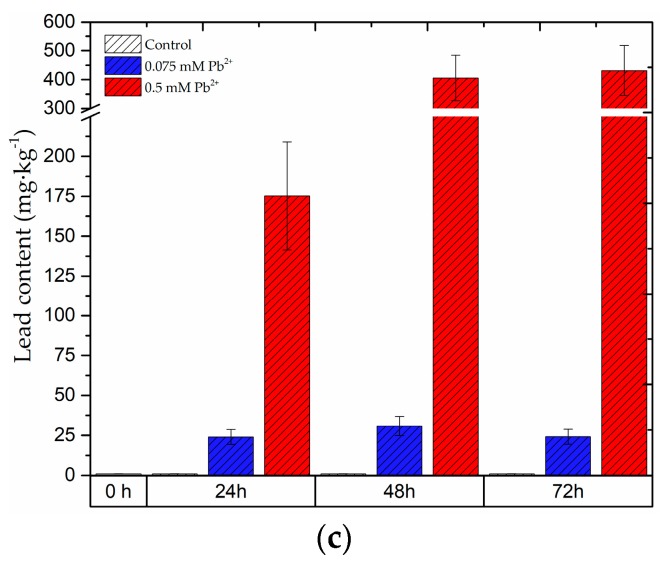
Lead contents in roots (**a**) and leaves (**b**) of pea seedlings and in bodies of pea aphids (**c**). The data were obtained in three independent experiments and statistically analysed using ANOVA (*p*-values at α = 0.05). Hypotheses on the equality of means were verified by the two-sample *t*-test. To account for multiple testing, we used the Bonferroni correction (statistically significant differences are shown in Table S1, S2).

Analysis of lead content in pea seedlings revealed that this heavy metal was accumulated in large quantities in the roots of pea seedlings. Already at 0 h of the experiments, i.e., after 4 days from the administration of lead, accumulation of this element was observed both in the roots of seedlings growing on the medium with 0.5 mM Pb(NO_3_)_2_ and 0.075 mM Pb(NO_3_)_2_ (Figure 10, Table S1). However, lead concentration in the tissue of roots cultured on the medium with the high lead concentration was over 3.5 times higher than in the tissues cultured on the medium with its low concentration. At subsequent time points of the experiment, lead content in roots growing on the medium with 0.5 mM Pb(NO_3_)_2_ increased as compared to that in roots at 0 h. Thus, the following values were recorded: at 0 h 18,569.94 mg·kg^−1^, at 24 h 24,571.18 mg·kg^−1^, at 48 h 24,911.73 mg·kg^−1^ and at 72 h 27,750.66 mg·kg^−1^, respectively. In roots of pea seedlings growing on the medium with lead at the high and the low concentrations and infested by *A. pisum* (0.075 mM Pb^2+^ + aphids and 0.5 mM Pb^2+^ + aphids variants), the content of lead increased slightly, but only at 24 hpi, while at the next time point it was lower than in the 0.075 mM Pb^2+^ and 0.5 mM Pb^2+^ variants. Furthermore, at the administration of the lower lead concentration to the medium its contents in the seedling roots were as follows: at 0 h 5027.8 mg·kg^−1^, 24 h 4343.16 mg·kg^−1^, 48 h 5589.32 mg·kg^−1^ and 72 h 5202.08 mg·kg^−1^. Administration of lead at 0.5 mM Pb(NO_3_)_2_ caused an increased lead content in the seedling leaves. Lead contents in leaves of the 0.5 mM Pb^2+^ variant were as follows: at 0 h 19.64158 mg·kg^−1^, 24 h 30.04383 mg·kg^−1^, 48 h 23.7783 mg·kg^−1^ and 72 h 37.78 mg·kg^−1^. In turn, lead contents in leaves of the 0.075 mM Pb^2+^ variant at 0 h was 1.89381 mg·kg^−1^, at 24 h 1.72839 mg·kg^−1^, 48 h 2.49095 mg·kg^−1^ and 72 h 5.05971 mg·kg^−1^, respectively. Additionally, pea aphid infestation caused increased lead contents in leaves of pea seedlings at all time points after infestation. The highest content was recorded at 72 hpi in pea seedlings growing on the medium with 0.5 mM Pb(NO_3_)_2_ and infested by *A. pisum*, amounting to 67.28 mg·kg^−1^. Additionally, as it was mentioned above, pea aphid feeding stimulated lead uptake and increased lead contents also in leaves of pea seedlings in the 0.075 mM Pb^2+^ + aphids variant, i.e., at 24 hpi it was 2.84 times, at 48 h 2.87 times and at 72 h 2.64 times in relation to lead contents in leaves of the 0.075 mM Pb^2+^ variant, respectively. Moreover, it should be mentioned here that at the beginning of the experiment (i.e., 4 days after lead administration), lead concentration in leaves of pea seedlings growing on the medium with 0.5 mM Pb(NO_3_)_2_ was 10 times higher than in leaves of seedlings growing on the medium with 0.075 mM Pb(NO_3_)_2_.

Lead content in bodies of pea aphids increased with infestation time, i.e., in the period from 24 to 72 hpi (Figure 10c, Table S2). The highest lead content was recorded at 72 hpi in bodies of pea aphids, ranging from 175.1867 mg·kg^−1^ to 431.62322 mg·kg^−1^. In turn, in bodies of pea aphids feeding on pea seedlings growing on the medium with 0.075 mM Pb(NO_3_)_2_ the content of lead was markedly lower than in the bodies of pea aphids feeding on pea seedlings growing on the medium with 0.5 mM Pb(NO_3_)_2_, with the levels ranging from 24.0655 mg·kg^−1^ to 30.8712 mg·kg^−1^, respectively.

## 3. Discussion

An important goal from the point of view of ecology is to study interactions between biotic and abiotic stresses at different levels of biological organisation [46]. Exposure of plants to one stress alters their metabolic status, which can influence the intensity or ability to adaptively respond to a subsequent stress. The results obtained within this study show for the first time the relationship between different concentrations of lead in the medium in terms of their effect on the growth of pea seedlings, pea aphid infestation and defensive response of plants manifested in the generation of signalling molecules. Moreover, it is a novel finding to show an enhanced generation of phytohormones such as SA and ABA in roots and leaves of pea seedlings and induction of biosynthesis of secondary metabolites, i.e., flavonoids, in the context of the effect of lead and the cross-talk of lead and a phytophage with the piercing-sucking mouthpart, i.e., *A. pisum*. Presently more evidence needs to be provided for the simultaneous impact of environmental factors on the defense strategy of the plants, i.e., to show the relationship of an abiotic stress factor (heavy metal) and plant defenses induced by a biotic stress factor (aphids). Also, the results within this research work provide new insights concerning the phenomenon of hormesis: the abiotic factor (lead)—plant (signalling molecules and flavonoids, including pisatin)—herbivore (the insect with a piercing-sucking mouthpart).

We demonstrated that after administration of lead at a high concentration in the substrate at 0.5 mM Pb(NO_3_)_2_, a high concentration of this element was observed first in the roots and then in the leaves of pea seedlings. Also, lead was downloaded by pea aphids, especially in large amounts when aphids were feeding on pea seedlings growing on the medium with 0.5 mM Pb(NO_3_)_2_. In turn, at the low concentration of 0.075 mM Pb(NO_3_)_2_, this element was mainly accumulated in the roots, while only small amounts appeared in the leaves and next in bodies of *A. pisum*, which was in line with our assumption. At a low concentration of lead deposited mainly in roots the consequence was an enhanced metabolic status of pea seedlings, which elicited defense mechanisms of the leaves to the biotic stress factor. Therefore, after 4 days from the administration of lead, in 0 h-leaves of the 0.075 mM Pb(NO_3_)_2_ variant an increased level of signalling molecules such as SA was noted. Statistical analysis showed significant differences between levels of SA or SAG in leaves of the 0.075 mM Pb(NO_3_)_2_ variant and the control variant. However, the content of an isoflavonoid phytoalexin, pisatin, in leaves with a low lead level at this point time was not increased in relation to the control leaves, while an increase was observed only for some glucosides and the PAL activity, an enzyme initiating phenylpropanoid metabolism. The increase in pisatin levels in the above-mentioned leaves was found at 48 and 72 hpi as a result of lead or lead and *A. pisum* interactions; however, it was significantly lower than in the case of the impact of lead with the high concentration. It should be emphasized that the different concentrations of the abiotic factor in the medium caused the different defensive response on multiple levels. In addition, direct contact of the stress factor (lead) with the roots caused a stronger defense response of the roots in comparison to the leaves. An interesting observation was noted after pea aphid feeding on pea seedlings cultured on the medium with lead at both concentrations. It was shown that *A. pisum* infestation modulated molecular and metabolic responses in the pea seedlings both with the high and low lead concentration. These changes included both the roots and the leaves, which indicates systemic signalling from the leaves to the roots, triggered by *A. pisum* feeding on the pea seedlings. The analysis of generation intensity for TSA (SA and SAG) and ABA in the roots and the leaves of pea seedlings growing on the medium with lead and during the treatment of both lead and *A. pisum* shows that SA is a molecule intensively stimulated both by lead alone and the cross-talk of lead and *A. pisum*. Also, a strong SA accumulation was found in the roots and the leaves during *A. pisum* feeding infestation alone, while higher levels of TSA were detected in the roots rather than the leaves. In turn, an opposite result was obtained for ABA, as a higher level of this molecule was found in the leaves than in the roots. Moreover, in the leaves the very strong TSA accumulation was a result of the amplification of the signal coming from lead and *A. pisum*, with the response being stronger at the high lead concentration than at the low concentration. In the roots a similar effect for SA was observed, but it was visible only at 72 hpi. Moreover, it should be stressed here that for ABA a slightly different trend was recorded. Lead treatment stimulated ABA accumulation most strongly. The amplification of the signal from lead and *A. pisum* was only observed at 72 hpi in the leaves with a high lead content and at 24 hpi in the leaves with a low lead content, respectively.

In the published literature the important role of SA and ABA was demonstrated both in responses to biotic and abiotic stresses, as well as in the development of plants ([47,48,49,50,51] and references in this article). These phytohormones play a crucial role in adaptive responses to stresses. SA as a signal molecule induces plant defense responses to biotic stress factors, including insect pests ([52,53] and references in this article). Besides, SA has been assigned roles in improved plant tolerance to stress via SA-mediated control of major metabolic processes in plants [51]. SA treatments induced different physiological and biochemical processes (e.g., enhanced the level of oxidative stress, the expression of genes involved in flavonoid metabolism and the amount of non-enzymatic antioxidants) in the leaves and roots of plants; however, the response of these organs varied [10]. Moreover, salicylates are molecules that may activate defense genes in plant response to stress. Upon intensification of these signals, an increase in the synthesis of allelochemicals and defence proteins was triggered [54], providing protection to the plants. It was also suggested that SA antagonises the oxidative damaging effects of lead both directly by activating the antioxidative enzymatic system and indirectly by decreasing lead uptake from soil [55]. Additionally, Chen et al. [56] demonstrated that SA alleviates lead-induced membrane disruptions and plays a positive role in rice seedlings protecting them against toxicity. Additionally, it has been revealed that lead causes an increase in endogenous free SA levels in all organs of *Zygophyllum fabago* [57]. The application of SA on wheat induced plant resistance to aphids [58]. El-Khawas [59] reported that priming plants with SA induced resistance of crop plants against herbivores, showing it to be successful in *Pisum sativum* against the leafminer *Liriomyza trifolii*. Therefore, an increase in the SA level was often observed in plant response to abiotic and biotic stresses, including aphid infestation [8,60]. Additionally, there are reports concerning the significance of SA in the minimisation of impacts of heavy metals, including lead [56,61,62,63]. It is believed that phytohormones such as SA and ABA can be promising compounds for the reduction of plant sensitivity to stresses, which may be important for agriculture [10,64]. The results of our experiments have shown that lead treatment and aphid attack seem to affect SA and pisatin accumulation synergistically. Lead treatment caused stimulation of PAL activity in leaves of seedlings after 4 days (0 h). Additionally, the cross-talk of these two stressors, i.e., lead at 0.5 mM Pb(NO_3_)_2_ and *A. pisum* significantly induced the activity of the enzyme in leaves of the 0.5 mM Pb(NO_3_)_2_ + aphids variant. What is more, amplification of the signal induced by the cross-talk of lead and *A. pisum* attack strongly upregulated the expression of PAL and CHS genes in the leaves of pea seedlings; it was stronger for CHS than for PAL. On the other hand, a synergistic positive role with jasmonate-induced defenses against herbivores and an antagonistic role with salicylate-based resistance to some pathogens was observed in the case of plant responses to water and salt stress by Thaler and Bostock [46]. Besides, the role of ABA as a central regulator of abiotic stress response in plants has been proven in numerous studies [65,66,67]. ABA concentration in plant tissues is known to increase when plants are exposed to heavy metals, suggesting an involvement of this phytohormone in the induction of protective mechanisms against heavy metal toxicity [68,69,70]. Stroiński et al. [71] reported that ABA was required in the transduction of the cadmium (Cd) signal to potato roots. For example, cadmium treatment leads to increased endogenous ABA levels in roots of *Typha latifolia* and *Phragmites australis* [72], in potato tubers [73] as well as rice plants [74]. The same effect was verified in several other studies. When mercury (Hg), Cd and copper (Cu) solutions were applied to wheat seeds during germination, ABA levels increased [75]. In cucumbers, seed germination decreased and ABA content increased under Cu^2+^ and zinc (Zn^2+^) stress [76]. Similarly, increased amounts of ABA were detected in germinating chickpea (*Cicer arietinum*) seeds under lead (Pb) toxicity conditions [77], as well as crowberries (*Empetrum nigrum*) exposed to Cu and nickel (Ni) [78]. Strong SA and ABA generation found in *P. sativum* L. cv. Cysterski seedlings during the influence of lead and the cross-talk of lead and *A. pisum*, especially at a high lead concentration, may indicate the involvement of these molecules in defensive response. However, as it was mentioned above, lead and aphid attack seem to affect SA and pisatin accumulation synergistically. In the case of ABA, this dependence was demonstrated only at 72 hpi. In the published literature some reports have shown that ABA was involved in the modulation of the phenylpropanoid pathway in berry skins [79,80]. Exogenous applications of ABA to red grape berries caused an increase in the flavonoid content [79,80,81,82]. In turn, in stressed plants ABA treatment regulated the expression of genes encoding enzymes of the flavonoid biosynthesis pathway [83]. In the case of our experiments, apart from an enhanced generation of signalling molecules under stress conditions, upregulation of phenylpropanoid metabolism genes, a strong accumulation of flavonoids and a strong stimulation of the activity of β-glucosidase were also recorded. The upregulation of CHS mRNA and pisatin accumulation in leaves of pea seedlings infested by the pea aphid at varying population sizes was observed in our previous studies [20]. We demonstrated also the participation of another signal molecule such as nitric oxide (NO) in pisatin accumulation and upregulation of the relative mRNA levels for PAL in leaves pretreated with NO donors (GSNO and SNP), both infested and non-infested tissues [84]. Moreover, there are reports concerning a transcriptional and posttranscriptional control of PAL expression under heavy metals stress [36]. The Pb induced PAL mRNA levels in legume plants exposed to heavy metals stress. 

Additionally, our results show also a significant accumulation of flavonoid and isoflavonoid glycosides in response to lead alone and to the cross-talk of lead and the phytophage. Higher levels of these metabolites were recorded in the roots than in the leaves. As it was already mentioned above, the level of glycosides was also higher in the organs of pea seedlings growing with a high lead concentration in comparison to seedlings cultured with a low lead concentration. Generally, the dominant trend was associated with reduced levels of the metabolites in tissues affected by lead and aphids in comparison to those affected by lead treatment. In contrast, the reduction of flavonoid and isoflavonoid glycosides was observed mainly at the administration of the toxic dose of lead in the medium and *A. pisum* infestation in relation to their levels in leaves of the 0.5 mM Pb^2+^ variant. Additionally, the reduction of these metabolites was stronger in the roots than in the leaves. The reduction of glycosides accompanied a high activity of β-glucosidase in pea seedlings growing with lead, or the variant with lead and infested by aphids, or only that with the attack by aphids. An increase in the activity of this enzyme may be related with the release of metabolites toxic for aphids or participation in the reconstruction of cell wall damage and the strengthening of its structure [85]. In turn, in our experiments reduction of the enzyme activity at 72 hpi in both leaves and roots may be due to the effect of *A. pisum* effectors that may block defense mechanisms of the host plant, including hydrolysis of glucosides to free aglycones. Many of the isoflavonoids and flavonoids have reactive free hydroxyl groups which are biologically active [86]. A diverse group of phytoalexins, particularly their chemical diversity, the main biosynthetic pathways and their regulatory mechanisms, phytoalexin gene transfer in plants and their role as antibiotic agents, were presented by Jeandet [87]. Also, information has also been provided concerning modulation of phytoalexin levels through engineering of plant hormones, defence-related markers or elicitors [88]. Our earlier research results [20] revealed also subcellular location of flavonoids using confocal microscopy in leaves of pea seedlings infested by aphids [20]. Furthermore, Adrian et al. [89] for the first time showed that aluminum chloride can act as a potent elicitor of resveratrol synthesis in grapevine leaves. Strong evidence was provided that a metallic salt can act as a direct inducer of phytoalexin response in grapevines.

Additionally, very interesting results of these studies showed that the toxic concentration of lead strongly inhibited growth of pea seedlings (root length in the 0.5 mM Pb^2+^ variant was about 2 times shorter than in the control and 0.075 mM Pb^2+^ variants), while the low concentration of 0.075 mM Pb(NO_3_)_2_ stimulated slightly growth, especially the shoots of seedlings, thus indicating the hormesis effect. Extremely interesting are results of quantitative analysis of metabolites in the roots and leaves of pea seedlings. Here the Principal Component Analysis (PCA) of the positive and negative ions MS revealed that groups ions in the control, + aphids, 0.075 mM Pb^2+^, 0.075 mM Pb^2+^ + aphids variants are clustered close. There may be a link between growth and the metabolome of plants from these variants. In turn, groups of the 0.5 mM Pb^2+^ and 0.5 mM Pb^2+^ + aphids variants were distinct in relation to the other.

It is known that at elevated concentrations heavy metals negatively affect morphology, physiology and biochemistry of plants [90,91,92,93,94]. The inhibition growth of lupin roots was observed in response to heavy metals (Pb, Cd and Cu), which was accompanied by an increased synthesis and accumulation of a 16 kDa polypeptide [95]. In turn, Rucińska et al. [96] reported that at higher lead concentrations the formation of both free radicals and reactive oxygen species is beyond the capacity of the antioxidant system, which in turn may contribute to reduced root growth. Lead can induce several morphological, physiological and biochemical dysfunctions in plants, such as a decrease in seed germination, plant growth, chlorophyll production, etc., while it also causes lipid peroxidation, oxidative stress and DNA damage [97]. A decrease was also recorded in dry weight of both roots and shoots [98,99]. Lead can reduce 50% of growth when applied at a concentration of 1000 mg/L in *Eichhornia crassipes* (water hyacinth). Also, an increase in lead concentration caused a decrease in chlorophyll content and an increase in the activity of antioxidative enzymes [100]. The results of the studies showed that Pb is a highly toxic element that can be accumulated in the cell wall, the cell membrane, vacuoles, mitochondria and peroxisomes. Małecka et al. [101]. Krzesłowska et al. [102] reported that plant cell wall (CW) remodeling, in particular formation of CW thickenings (CWTs) abundant in low-methylesterified pectins (pectin epitope JIM5 - JIM5-P) able to bind metal ions, including Pb, certainly increases the CW capacity for toxic metal ion binding and compartmentalisation. Rabęda et al. [103] reported that binding trace metals by JIM5-P may lead to a reduction of cell elongation and plant growth inhibition. Moreover, results of our research highlighted lead-induced hormetic growth response. Hormesis and the paradoxical effect of Pb at a low concentration on growth of wheat seedlings was revealed by Erofeeva et al. [104], but it was noted for Cd. In turn, a hormetic response of *Lonicera japonica* induced by Cd at low concentrations (≤10 mg·kg^−1^) closely related to the increase in net photosynthesis was demonstrated by Jia et al. [105]. Numerous vivid discussions concerning the importance of hormesis responses in toxicology and risk assessment may be followed in scientific papers and in the media, particularly during the last 15 years. It is assumed that hormesis is an adaptive response to stress. Recently, in a further attempt to convert the classical descriptive term of hormesis into a mechanistically based concept, it was proposed to consider hormesis as an adaptive response that is “providing a quantitative estimate of biological plasticity” [5]. In the published literature some reports indicate that herbivorous insects feeding on contaminated plants may suffer from developmental disorders manifested e.g., in an alteration of some life history traits, such as changes in morphology. When specimens of the cabbage aphid *Brevicoryne brassicae* L. were reared on lead contaminated host plants, they were smaller and showed a considerable degree of morphological asymmetry in comparison to aphids on non-contaminated plants [106,107]. Moreover, Poschenrieder et al. [2] reported that heavy metals in plants may affect herbivores, e.g., by acting as an antifeedant or a systemic plant pesticide, or by fortifying their antioxidant defenses. 

In summary, this study supplies additional new information on the responses of pea seedlings to cross-talk of a heavy metal, lead, and aphid attacks. We have shown a relationship between SA and ABA-mediated signalling and their impact on the accumulation of flavonoids, including an isoflavonoid phytoalexin, pisatin. Also, we have demonstrated changes in the expression of key enzymes of the phenylpropanoid pathway (PAL and CHS) and the activity of β-glucosidase, an enzyme hydrolysing flavonoid glucosides to free aglycones, in the context of the cross-talk of lead and aphid attack. Moreover, our results demonstrate that a response of the plant to one stress with varying severity influences its response to other stresses.

In the future it is necessary to provide more evidence for the cross-tolerance phenomenon. Moreover, it is interesting to examine the interactions between hormone-signaling pathways and their impact on defence responses of plants and the ecological pattern of association of stresses.

## 4. Materials and Methods 

### 4.1. Plant Material and Growth Conditions

Pea (*Pisum sativum* L. cv. Cysterski) seeds of the S-elite class were used in the experiments, these were obtained from the Plant Breeding Company at Tulce near Poznan in Poland. Surface-sterilisation of seeds was performed as described by Mai et al. [8] and Morkunas et al. [20]. After 6 h of imbibition the seeds were transferred onto filter paper (in Petri dishes) and immersed in a small amount of water in order to support further absorption. After a subsequent 66 h the seed coats were removed from the germinating seeds. Next the germinating seeds (35 pieces) were transferred to hydroponic grow boxes containing Hoagland medium (the control). Hydroponic boxes were covered with dark foil to mimic soil conditions. The experiments were conducted on seedlings of pea (*P. sativum*). Pea seedlings during the first four days were cultured in hydroponic cultures on the Hoagland medium in a phytotrone at 22–23 °C, 65% relative humidity, and light intensity of 130−150 μM photons m^−2^s^−1^ with the 14/10 h (light/dark) photoperiod. Next, on the fifth day the medium was replaced in all the hydroponic variants and lead was added to the medium at 0.075 Pb(NO_3_)_2_ and 0.5 mM Pb(NO_3_)_2_. After 4 days from the administration of lead, pea aphids were transferred onto pea seedlings. The following experimental variants were used: the control pea seedlings cultured with no addition of lead and not colonised by pea aphids (*Acyrthosiphon pisum*), pea seedlings growing on the Hoagland medium with different concentrations of lead ions, i.e., 0.075 mM Pb(NO_3_)_2_ and 0.5 mM Pb(NO_3_)_2_ and pea seedlings growing on the Hoagland medium with different concentrations of lead ions and colonised by pea aphids *A. pisum*, pea seedlings growing on the Hoagland medium colonised by pea aphids *A. pisum*. Samples for analyses were collected 4 days after lead administration and prior to transferring aphids onto pea seedlings (at 0 h), and next after 24, 48, 72 h of the action of the two stress factors, i.e., lead and *A. pisum*. Hydroponic cultures were aerated with an aeration system. Pea seedlings, both the control and growing in the presence of lead ions and seedlings growing in the presence of lead ions for colonisation by *A. pisum*, were cultured in glass aquariums (30 cm × 22 cm × 28 cm) and protected with gauze. Main experimental treatments with the insects comprised leaves of pea seedlings, while analyses were also performed for roots, in order to obtain comprehensive information concerning dependencies of root-leaves after the addition of lead to the medium at different concentrations. The experiments were conducted only using adult insects.

### 4.2. Aphids and Infestation Experiment 

*Acyrthosiphon pisum* (Harris), originally cultured and supplied by the Department of Entomology, the Poznań University of Life Sciences, Poland, was reared on *Pisum sativum* L. in a growth chamber under conditions as specified above. On day 11 of culture pea seedlings were infested with 20 apterous adult females of *A. pisum*. The aphid populations were monitored throughout all the experiments [108]. The control pea seedlings were cultured with no addition of lead and not colonised by pea aphids (*Acyrthosiphon pisum*).

### 4.3. Detection of Salicylic Acid (SA)

Salicylic acid was extracted and quantified following the HPLC method, as previously described by Yalpani et al. [109] and modified by Mai et al. [8]. Frozen roots and leaves were ground in liquid nitrogen to a homogenous powder, from which approximately 0.50 g was taken for analysis. Salicylic acid was extracted twice with methanol (90% followed by a straight solvent), strongly stirred and centrifuged at 12,000 × g for 10 min at 4 °C. The resultant supernatant was divided into two equal batches and the solvent was evaporated to dryness under a stream of nitrogen. Dry extract was dissolved in 5% trichloroacetic acid (TCA), and after centrifugation free salicylic acid (SA) was extracted three times with the extractive organic mixture of ethyl acetate:cyclopentane:isopropanol (100:99:1; *v*/*v*/*v*). In order to determine free and glucoside bound salicylic acid (TSA), 40 units of β-glucosidase in Na-acetate buffer (100 mM, pH 5.2) were added to the second part of the dry extract and incubated for 90 min at 37 °C. Lyophilised β-glucosidase from almonds (Sigma-Aldrich) was used; according to the manufacturer’s specification one unit liberates 1.0 μmole of glucose from salicin per min at pH 5.0 and 37 °C. The reaction was terminated by the addition of 5% TCA and salicylic acid was extracted as described above. After solvent evaporation, the dry residue was dissolved in a mobile phase (200 mM K-acetate buffer, pH 5.0 and 0.5 mM ethylenedinitrilotetraacetic acid, EDTA) and analyzed by HPLC coupled with fluorometric detection in a Waters Company chromatograph (Milford, MA, USA) composed of a 2699 Separation Module Alliance and 2475 Multi- Fluorescence Detector. Chromatographic separation was obtained in a Spherisorb ODS2 WATERS Company column (4.6 mm × 10 mm, 3 µm). Detection parameters were λ=295 nm for excitation and 405 nm for emission. The content of glucoside bound salicylic acid (SAG) was calculated as the difference between total and free salicylic acid (TSA-SA) and the results were expressed as nanograms per gram of fresh weight material (ng g^−1^ FW).

### 4.4. Detection of Abscisic Acid (ABA)

For ABA measurement samples consisting of approx. 1 g frozen tissue were homogenised in liquid nitrogen and extracted twice with 20 mL 80% (*v*/*v*) methanol containing 20 mg/L butylated hydroxytoluene. Subsequently 100 ng [^2^H_6_]ABA were added. The methanol fraction was removed under reduced pressure and the aqueous phase was acidified to pH 2.0 with 12 M HCl and centrifuged at 10,000× *g* for 15 min to remove chlorophyll. The supernatant was partitioned three times against ethyl acetate and dried under vacuum. The dry residue was dissolved in 5 mL 1 M formic acid (FA) and loaded on a Discovery^®^ DSC−18 SPE cartridge (Supelco Inc., Bellefonte, PA, USA) pre-conditioned with 4 mL MeOH and allowed to equilibrate with 4.0 mL of 1 M FA. The column was then washed with 4 mL 1 M FA, 20% of methanol in 1M FA and finally target phytohormones were eluted with 4 mL of 80% methanol in water. The eluate was evaporated and further purified by HPLC using a SUPELCOSIL ABZ + PLUS column (250 mm × 4.5 mm, 5 µm article size; Supelco). The samples were reconstituted in 130 µL of 20 % methanol and chromatographed with a linear gradient of 20–80% methanol in 0.1 M formic acid for 20 min, flow rate 1.0 mL/min at 22 °C. The fractions collected at 12.5 ± 0.5 min were evaporated to dryness, methylated with diazomethane, dissolved in 30 µL of methanol and analyzed by GC/MS–SIM (Auto-System XL coupled to a TurboMass, Perkin-Elmer, Walthman, MA, USA) using a MDN-5 column (30 m × 0.25 mm, 0.25 µm phase thickness, Supelco). The GC temperature program was 60 °C for 1 min, 60–250 °C at 10 °C/min, flow rate 1.5 mL/min, injection port was 280 °C and electron potential 70 eV. The retention times of ABA and [^2^H_6_] ABA were 14.07 and 14.3 min, respectively. GC/MS–SIM was performed by monitoring *m*/*z* 190 for endogenous ABA and 194 for [^2^H_6_] ABA, 130 for endogenous IAA and 132 [^2^H_2_] IAA according to the method described by Vine et al. [110].

### 4.5. Analysis of Flavonoids

#### 4.5.1. Isolation of Phenolic Compounds

Plant material, previously frozen at −80 °C was homogenised in 80% methanol (20 mL·g^−1^·FW) and sonicated for 3 min in a VirTis VirSonic 60 sonicator [111]. The suspension was filtered through a Büchner funnel and concentrated under vacuum at 40 °C. Plant extract samples for LC analyses were prepared from 0.5 g FW pea tissue. The samples were purified and concentrated by solid-phase extraction on cartridges containing a cation exchanger and RP C−18 silica gel (Alltech, Carnforth, UK) used in tandem, according to the method proposed by Stobiecki et al. [112].

#### 4.5.2. Liquid Chromatography–Mass Spectrometry (LC/UV/ESI/MS/MS)

Plant extract samples were analysed using a Waters UPLC Acquity system coupled with a micrOToF-Q mass spectrometer (Bruker Daltonics, Bremen, Germany). An Agilent Poroshell RP-C18 column (100 mm × 2.1 mm; 2.7 µm) was used. During LC analyses elution was performed using two solvent mixtures: A (95% H_2_O, 4.5% acetonitrile, 0.5% acetic acid; *v*/*v*/*v*) and B (95% acetonitrile, 4.5% H_2_O, 0.5% acetic acid; *v*/*v*/*v*). Elution steps were as follows: 0–5 min 10%–30% B, 5–12 min isocratic at 30% B, 12–13 min linear gradient up to 95% of B and 13–15 min isocratic at 95% of B. Pisatin and flavonols were identified by comparing their retention times and mass spectra with the data from respective standards. The micrOToF-Q mass spectrometer consisted of an ESI source operating at a voltage of ±4.5 kV, nebulisation with nitrogen at 1.2 bar and dry gas flow of 8.0 L/min at 220 °C. The instrument was operated using the micrOTOF Control program version 2.3 and data were analysed using the Bruker Data Analysis ver. 4 package. Targeted MS/MS experiments were performed using a collision energy ranging from 10 to 25 eV, depending on the molecular masses of the compounds. The instrument operated at a resolution of minimum 15,000 full widths at half maximum.

#### 4.5.3. Quantitative Analysis of Metabolites

For targeted quantitative analysis, the extracted ion chromatogram traces were used, with peaks plotted for exact monoisotopic masses of compounds. Such traces were prepared for each compound earlier and identified based on their MS2 spectra. For calibration purposes, *p*-hydroxybenzoic acid was added to each analysed sample as the internal standard at a final concentration of 125 µM (LC retention time and MS spectra did not interfere with those of the studied compounds). Total quantitative analysis was conducted based on all signals acquired in the positive MS mode using the Profile Analysis 2.1 software (Bruker Daltonics). Multivariate analyses were carried out by the unsupervised principal component analysis (PCA).

### 4.6. Total RNA Extraction and Semiquantitative RT-PCR Analysis

Pea seedling leaves (0.50 g) were frozen in liquid nitrogen and ground with a mortar and pestle in the presence of liquid nitrogen. For RT-PCR analyses of the target gene, total RNA was isolated from 45 mg tissue using the SV Total RNA Isolation System (Promega, Manheim, Germany) according to the recommendations of the manufacturer [84,113]. The RNA level in samples was assayed spectrophotometrically at 260 nm. The A_260_/A_280_ ratio varied from 1.8 to 2.0. The cDNA samples for RT-PCR experiments were synthesised from 1 μg of total RNA and oligo (dT)_18_ primers using the High Capacity cDNA Reverse Transcription Kit (Applied Biosystems, Life Technologies Polska, Warszawa, Poland). One µL of the each cDNA was used as a template for the PCR reaction with specific PCR primers. Thermal cycling conditions in the PAL, CHS and actin genes expression assay consisted of an initial denaturation at 95 °C for 5 min, followed by 25 cycles at 95 °C for 30 s, 58 °C for 30 s and 72 °C for 45 s. The PCR products were analysed by agarose gel electrophoresis (1.5%). The specific primers were used for PCR reactions: PAL F 5’-GAGCAGCACAACCAAGATG-3′ and PAL R 5’-CTCCCTCTCAATTGACTTGG-3’, CHS F 5’-AGGTCTTCGCCCATATGTGAA-3’ and CHSR 5’-GTGCTTGTCCAACAAGACTGT-3’. The fragment of *Pisum sativum* actin (HQ231775) coding sequence was amplified as a reference gene using actin F 5′-GCATTGTAGGTCGTCCTCG-3′ and actin R 5′-TGTGCCTCATCACCAACATAT-3′ primers.

### 4.7. Extraction and Assay of β 1,3-Glucosidase Activity

The activity of β 1,3-glucosidase (EC 3.2.1.21) was determined spectrophotometrically (Lambda 15 UV-Vis spectrophotometer, Perkin Elmer, Norwalk, CT, USA) applying the method proposed by Nichols et al. [114] and modified by Morkunas et al. [115]. Leaves and roots of pea seedlings (500 mg) were ground at 4 °C in 0.05 M phosphate buffer of pH 7.0 and 1% polyvinylpyrrolidone (PVP). The enzyme activity was determined in the supernatant obtained after centrifugation at 15,000× *g* for 20 min. The mixture containing 0.2 mL phosphate buffer (0.05 M, pH 7.0), 0.2 mL extract and 0.2 mL 4-nitrophenyl-β-d-glucopyranoside as substrate (2 mg·mL^−1^) was incubated for one hour at 35 °C. Afterwards, 0.6 mL 0.2 M Na_2_CO_3_ was added. The formation of *p*-nitrophenol (*p*-NP) was followed at 400 nm. The activity was measured in three replications and expressed as μM *p*-nitrophenol mg^−1^·protein·h^−1^.

### 4.8. Extraction and Assay of Phenylalanine Ammonialyase (PAL) Activity

The activity of PAL (EC 4.3.1.24) was determined with a modified method of Cahill and McComb [116] as modified by Morkunas et al. [43]. The amount of 0.50 g of frozen leaves was homogenised at 4 °C with a mortar and pestle in 4 mL of 100 mM Tris–HCl buffer (pH 8.9) containing 5 mM mercaptoethanol, and 0.050 g PVP. Afterwards the homogenate was centrifuged at 12,000× g for 20 min at 4 °C. The supernatant was used for enzyme analyses. The reaction mixtures contained 0.50 mL of 20 mM borate buffer (pH 8.9), 0.50 mL of 10 mM l-phenylalanine, and 0.50 mL extract in a total volume of 1.5 mL. A sample without the substrate l-phenylalanine was used as a blank. The reaction proceeded for 24 h at 30 °C and was interrupted by the addition of 1.5 mL 2 N HCl. PAL activity was measured by the change of absorbance at 290 nm due to the formation of *tra*ns-cinnamic acid using a Perkin Elmer Lambda 15 UV-Vis spectrophotometer. The activity of PAL is expressed as µM *trans*-cinnamic acid expressed per mg protein per hour (µM *trans*-cinnamic acid· mg^−1^protein h^−1^).

### 4.9. Determination of Lead Content

All reagents and standards were of at least analytical grade. All solutions were prepared using ultrapure water 18.2 MΩ·cm. Root and leaf samples of pea seedlings were rinsed with ultrapure water (CDRX-200, Polwater, Kraków, Poland) and air dried at room temperature. Air dried samples were then ground (Planetary Micro Mill Pulverisette 7 premium line, Fritsch, Idar-Oberstein, Germany) and oven dried at 70 °C before weighing. Subsamples (0.5 g) were digested in 10 mL 65% nitric acid (Suprapur, Merck, Darmstadt, Germany) using a closed vessel digestion microwave system (MARS 5, CEM Corp., Matthews, NC, USA). The digested solutions were diluted to 50 mL using ultrapure water. The aphid samples were washed and dried as described for the plant samples. 0.2 g subsamples of aphids were digested in 10 mL 65% nitric acid (Suprapur, Merck) at 80 °C until the solution became clear. The digested solutions were diluted to 25 mL using ultrapure water. The lead concentrations were determined by flame atomic absorption spectroscopy (FAAS) (SpectrAA 240FS, Varian, Palo Alto, CA, USA) and graphite furnace atomic absorption spectroscopy with the Zeeman correction (GFAAS) (SpectrAA 240Z, Varian). Certified reference materials were analysed to verify the accuracy and precision of the measurements. Observed concentrations for the certified INCT-MPH-2 mixed Polish herbs sample (Institute of Nuclear Chemistry and Technology, Poland) were 2.02 mg·kg^−1^ Pb (certified 2.16 ± 0.23 mg·kg^−1^) for plant samples and 1.99 mg·kg^−1^ Pb for aphid samples. The Laboratory is accredited according to ISO/IEC 17025.

### 4.10. Statistical Analysis

All determinations were conducted within three independent experiments. Additionally, three biological replicates per experimental variant were made for a given experiment. Analysis of variance (ANOVA) was used to verify the significance of means from independent experiments within a given experimental variant. The elementary comparisons between particular levels of the analysed factor in different times (independently) were tested using the two-sample *t*-test for equal means for all the observed traits. To account for multiple testing, we used the Bonferroni correction. Moreover, comparisons related to the following plant material variants, i.e., the control vs. the 0.075 mM Pb^2+^ variant; the control vs. 0.5 mM Pb^2+^ variant; the control vs. the +aphids variant; the control vs. 0.075 mM Pb^2+^+aphids variant; the control vs. 0.5 mM Pb^2+^+aphids variant;. 0.075 mM Pb^2+^ variant vs. 0.5 mM Pb^2+^ variant; 0.075 mM Pb^2+^ variant vs. 0.075 mM Pb^2+^+aphids variant; 0.5 mM Pb^2+^ variant vs. 0.5 mM Pb^2+^+aphids variant; 0.075 mM Pb^2+^+aphids variant vs. 0.5 mM Pb^2+^+aphids variant; +aphids variant vs. 0.075 mM Pb^2+^+aphids variant; +aphids variant vs. 0.5 mM Pb^2+^+aphids variant. In turn, comparisons related to the following aphid variants, i.e., the control vs. 0.075 mM Pb^2+^ variant; the control vs. 0.5 mM Pb^2+^ variant; 0.075 mM Pb^2+^ variant vs. 0.5 mM Pb^2+^ variant, respectively. The figures present data obtained as means of triplicates for each variant along with standard errors of mean (SE). All the analyses were conducted using the GenStat v. 17 statistical software package.

## 5. Conclusions

In the presented study we revealed that lead at various concentrations (low causing the hormesis effect vs. high causing the toxic effect) and the cross-talk of lead and *A. pisum* induced generation of SA and ABA in pea seedlings. Increased generation of these phytohormones strongly enhanced the biosynthesis of flavonoids, including a phytoalexin, pisatin. Strong generation of SA and ABA found in pea seedlings as a result of the influence of lead alone, especially at a lead high concentration, and the cross-talk of lead and *A. pisum* may indicate the involvement of these molecules in defensive responses, with these responses being stronger at exposure to a toxic lead dose rather than at a low dose of lead.

## Supplementary Materials

Supplementary materials are available online.

## Abbreviations

ABA	abscisic acid
ANOVA	analysis of variance
Cd	cadmium
CHS	chalcone synthase
EDTA	ethylenedinitrilotetraacetic acid
ESI	electrospray ionization
FW	fresh weight
g	gram
Glc	glucose
h	hour
FA	formic acid
GC-MS	gas chromatography/mass spectrometry
hpi	hour post infestation
HPLC	high-performance liquid chromatography
IAA	indole-3yl-acetic acid
LC/UV/ESI/MS/MS	Liquid chromatography/ultraviolet detection/electrospray–mass spectrometry (tandem mass spectrometry)
MS	mass spectrometry
MS2	second stage of mass spectrometry
PAL	phenylalanine ammonia-lyase
PCA	principial component analysis
Pb	lead
REACH	Registration, Evaluation, Authorisation and Restriction of Chemicals
RT-PCR	Reverse transcription polymerase chain reaction
SA	salicylic acid
SAG	salicylic acid glucoside
SIM	Selected ion monitoring
TCA	trichloroacetic acid
TSA	total salicylic acid
UV-Vis	ultraviolet-visible spectrophotometry

## References

[1] Maksymiec W. (2007). Signaling responses in plants to heavy metal stress. Acta Physiol. Plant..

[2] Poschenrieder Ch., Tolrà R., Barceló J. (2006). Can metal defend plants against biotic stress?. Trends Plant Sci..

[3] Rejeb K.B., Abdelly C., Savouré A. (2014). How reactive oxygen species and proline face stress together. Plant Physiol. Biochem..

[4] Fujita M., Fujita Y., Noutoshi Y., Takahashi F., Narusaka Y., Yamaguchi-Shinozaki K., Shinozaki K. (2006). Crosstalk between abiotic and biotic stress responses: A current view from the points of convergence in the stress signaling networks. Curr. Opin. Plant Biol..

[5] Poschenrieder Ch., Cabot C., Martos S., Gallego B., Barceló J. (2013). Do toxic ions induce hormesis in plants?. Plant Sci..

[6] Arimura G., Maffei M.E. (2010). Calcium and secondary CPK signaling in plants in response to herbivore attack. Biochem. Biophys. Res. Commun..

[7] Arimura G., Ozawa R., Maffei M.E. (2011). Recent advances in plant early signaling in response to herbivory. Int. J. Mol. Sci..

[8] Mai V.Ch., Drzewiecka K., Jeleń H., Narożna D., Rucińka-Sobkowiak R., Kęsy J., Floryszak-Wieczorek J., Gabryś B., Morkunas I. (2014). Differential induction of *Pisum sativum* defense signaling molecules in response to pea aphid infestation. Plant Sci..

[9] Jeandet P., Courot E., Clément Ch., Ricord S., Crouzet J., Aziz A., Cordelier S. (2017). Molecular engineering of phytoalexins in plants: Benefits and limitations for food and agriculture. J. Agric. Food Chem..

[10] Gondor O.K., Pál M., Darkó É., Janda T., Szalai G. (2016). Salicylic acid and sodium salicylate alleviate cadmium toxicity to different extents in maize (*Zea mays* L.). PLoS ONE.

[11] Lattanzio V., Arpaia S., Cardinali A., di Venere D., Linsalata V. (2000). Role of endogenous flavonoids in resistance mechanism of *Vigna* to aphids. J. Agric. Food Chem..

[12] Simmonds M.S.J. (2003). Flavonoid–insect interactions: Recent advances in our knowledge. Phytochem.

[13] Leitner M., Boland W., Mithӧfer A. (2005). Direct and indirect defences induced by piercing-sucking and chewing herbivores in *Medicago truncatula*. New Phytol..

[14] Gould K.S., Lister C., Andersen Ø.M., Markham K.R. (2006). Flavonoid functions in plants. Flavonoids: Chemistry, Biochemistry and Applications.

[15] Bernards M.A., Båstrup-Spohr L., Schaller A. (2008). Phenylpropanoid metabolism induced by wounding and insect herbivory. Induced Plant Resistance to Herbivory.

[16] Lev-Yadun S., Gould K.S., Gould K.S., Davies K.M., Winefield C. (2009). Role of anthocyanins in plant defense. Anthocyanins Biosynthesis, Functions and Applications.

[17] Mewis I., Khan M.A.M., Glawischnig E., Schreiner M., Ulrichs Ch. (2012). Water stress and aphid feeding differentially influence metabolite composition in *Arabidopsis thaliana* (L.). PLoS ONE.

[18] Prince D.C., Drurey C., Zipfel C., Hogenhout S.A. (2014). The leucine-rich repeat receptor-like kinase Brassinosteroid Insensitive1-Associated Kinase1 and the Cytochrome P450 PHYTOALEXIN DEFICIENT3 contribute to innate immunity to aphids in arabidopsis. Plant Physiol..

[19] Golan K., Sempruch C., Górska-Drabik E., Czerniewicz P., Łagowska B., Kot I., Kmieć K., Magierowicz K., Leszczyński B. (2017). Accumulation of amino acids and phenolic compounds in biochemical plants responses to feeding of two different herbivorous arthropod pests. Arthropod-Plant Interact..

[20] Morkunas I., Woźniak A., Formela M., Mai V.Ch., Marczak Ł., Narożna D., Borowiak-Sobkowiak B., Kühn Ch., Grimm B. (2015). Pea aphid infestation induces changes in flavonoids, antioxidative defence, soluble sugars and sugar transporter expression in leaves of pea seedlings. Protoplasma.

[21] Hart S., Kogan M., Paxton J.D. (1983). Effect of soybean phytoalexins on the herbivorus insects mexican bean bettle and soybean looper. J. Chem. Ecol..

[22] Russel G.B., Sutherland O.R.W., Hutchins R.F.N., Chrostmas P.E. (1978). Vestitol: A phytoalexin with insect feeding-deterrent activity. J. Chem. Ecol..

[23] Sutherland O., Russell G., Biggs D., Lane G. (1980). Insect feeding deterrent activity of phytoalexin isoflavonoids. Biochem. Syst. Ecol..

[24] Morimoto M., Kumeda S., Komai K. (2000). Insect antifeedant flavonoids from *Gnaphalium affin*. J. Agric. Food Chem..

[25] Onyilagha J.C., Lazorko J., Gruber M.Y., Soroka J.J., Erlandson M.A. (2004). Effect of flavonoids on feeding preference and development of the crucifer pest *Mamestra configurata* Walker. J. Chem. Ecol..

[26] Zhou D.S., Wang C.Z., Loon J.J.A. (2009). Chemosensory basis of behavioural plasticity in response to deterrent plant chemicals in the larva of the small cabbage white butterfly *Pieris rapae*. J. Insect Physiol..

[27] Goławska S., Łukasik I. (2012). Antifeedant activity of luteolin and genistein against the pea aphid, *Acyrthosiphon pisum*. J. Pest Sci..

[28] Simmonds M.S.J. (2001). Importance of flavonoids in insect-plant interactions: Feeding and oviposition. Phytochemistry.

[29] Ateyyat M., Abu-Romman S., Abu-Darwish M., Ghabeish I. (2012). Impact of flavonoids against woolly apple aphid, *Eriosoma lanigerum* (Hausmann) and its sole parasitoid, *Aphelinus mali* (Hald.). J. Agric. Sci..

[30] Diaz Napal G.N., Palacios S.M. (2015). Bioinsecticidal effect of flavonoids pinocembrin and quercetin against *Spodoptera frugiperda*. J. Pest Sci..

[31] Diaz J., Bernal A., Pomar F., Merino F. (2001). Induction of shikimate dehydrogenase and peroxidase in pepper (*Capsicum annuum* L.) seedling in response to copper stress and its relation to lignification. Plant Sci..

[32] Sakihama Y., Cohen M.F., Grace S.C., Yamasaki H. (2002). Plant phenolic antioxidant and prooxidant activities: Phenolics-induced oxidative damage mediated by metals in plants. Toxicology.

[33] Smeets K., Cuypers A., Lambrechts A., Semane B., Hoet P., Laere A.V., Vangorsveld J. (2005). Induction of oxidative stress and antioxidative mechanisms in *Phaseolus vulgaris* after Cd application. J. Plant Physiol. Biochem..

[34] Jahangir M., Abdel-Farid I.B., Kim H.K., Choi Y.H., Verpoorte R. (2009). Healthy and unhealthy plants: The effect of stress on the metabolism of Brassicaceae. Environ. Exp. Bot..

[35] Posmyk M.M., Kontek R., Janas K.M. (2009). Antioxidant enzymes activity and phenolic compounds content in red cabbage seedlings expose to copper stress. Ecotoxicol. Env. Saf..

[36] Pawlak-Sprada S., Arasimowicz-Jelonek M., Podgórska M., Deckert J. (2011). Activation of phenylpropanoid pathway in legume plants expose to heavy metals: Part I. Effects of cadmium and lead on phenylalanine ammonia-lyase gene expression, enzyme activity and lignin content. Acta Biochim. Pol..

[37] Rastgoo L., Alemzadeh A. (2011). Biochemical responses of Gouan (*Aeluropus littoralis*) to heavy metals stress. Aust. J. Crop Sci..

[38] Ashraf U., Kanu A.S., Deng Q., Mo Z., Pan S., Tian H., Tang X. (2017). Lead (Pb) toxicity; physio-biochemical mechanisms, grain yield, quality, and Pb distribution proportions in scented rice. Front. Plant Sci..

[39] Arshad M., Silvestre J., Pinelli E., Kallerhoff J., Kaemmerer M., Tarigo A., Shahid M., Guiresse M., Pradere P., Dumat C. (2008). A field study of lead phytoextraction by various scented Pelargonium cultivars. Chemosphere.

[40] Uzu G., Sobanska S., Sarret G., Munoz M., Dumat C. (2010). Foliar lead uptake by lettuce exposed to atmospheric fallouts. Environ. Sci. Technol..

[41] Krzywy I., Krzywy E., Pastuszak-Gabinowska M., Brodkiewicz A. (2010). Lead- is there something to be afraid of?. Ann. Acad. Med. Stetin..

[42] Pourrut B., Shahid M., Dumat C., Winterton P., Pinelli E., Whitacre D.M. (2011). Lead uptake, toxicity, and detoxification in plants. Reviews of Environmental Contamination and Toxicology.

[43] Morkunas I., Marczak Ł., Stachowiak J., Stobiecki M. (2005). Sucrose-induced lupine defense against *Fusarium oxysporum*: Sucrose-stimulated accumulation of isoflavonoids as a defense response of lupine to *Fusarium oxysporum*. Plant Physiol. Biochem..

[44] Chen Z., Zheng Z., Huang J., Lai Z., Fan B. (2009). Biosynthesis of salicylic acid in plants. Plant Signal. Behav..

[45] Liu Y., Liu H., Pan Q., Yang H., Zhan J., Huang W. (2009). The plasma membrane H^+^-ATPase is related to the development of salicylic acid-induced thermotolerance in pea leaves. Planta.

[46] Thaler J.S., Bostock R.M. (2004). Interactions between abscisic acid-mediated responses and plant resistance to pathogens and insects. Ecology.

[47] Metwally A., Finkermeier I., Georgi M., Dietz K.J. (2003). Salicylic acid alleviates the cadmium toxicity in barley seedlings. Plant Physiol..

[48] Horvath E., Szalai G., Janda T. (2007). Induction of abiotic stress tolerance by salicylic acid signalling. J. Plant Growth Reg..

[49] Vlot A.C., Dempsey D.A., Klessig D.F. (2009). Salicylic acid, a multifaceted hormone to combat disease. Annu. Rev. Phytopathol..

[50] Leng P., Yuan B., Guo Y., Chen P. (2014). The role of abscisic acid in fruit ripening and responses to abiotic stress. J. Exp. Bot..

[51] Khan M.I.R., Fatma M., Per T.S., Anjum N.A., Khan N.A. (2015). Salicylic acid-induced abiotic stress tolerance and underlying mechanisms in plants. Front. Plant Sci..

[52] Pickett J.A., Poppy G.M. (2001). Switching on plant genes by external chemical signals. Trends Plant Sci..

[53] Ton J., Flors V., Mauch-Mani B. (2009). The multifacede role of ABA in disease resistance. Trends Plant Sci..

[54] Li X., Schuler M.A., Berenbaum M.R. (2002). Jasmonate and salicylate induce expression of herbivore cytochrome P450 genes. Nature.

[55] Jing Y., He Z., Yang X. (2007). Role of soil rhizobacteria in phytoremediation of heavy metal contaminated soils. J. Zhejiang Univ. Sci. B.

[56] Chen J., Zhu C., Li L.P., Sun Z.Y. (2007). Effects of exogenous salicylic acid on growth and H_2_O_2_– metabolizing enzymes in rice seedlings under lead stress. J. Environ. Sci..

[57] López-Orenes A., Martinez-Pérez A., Calderón A.A., Ferrer M.A. (2014). Pb-induced response in *Zygophyllum fabago* plants are organ-dependent and modulated by salicylic acid. Plant Physiol. Biochem..

[58] Mahmoud E.M.F., Mahfouz H.M. (2015). Effects of salicylic acid elicitor against aphids on wheat and detection of infestation using infrared thermal imaging technique in Ismailia. Pestic. Phytomed. (Belgrade).

[59] El-Khawas S.A. (2012). Priming *Pisum sativum* with salicylic acid against the leafminer *Liriomyza trifolii*. Afr. J. Agric. Res..

[60] Janda T., Gondor O.K., Yordanova R., Szalai G., Pál M. (2014). Salicylic acid and photosynthesis: Signalling and effects. Acta Physiol. Plant..

[61] Krantev A., Yordanova R., Janda T., Szalai G., Popova L. (2008). Treatment with salicylic acid decreases the effect of cadmium on photosynthesis in maize plants. J. Plant Physiol..

[62] Zengin F. (2014). Exogenous treatment with salicylic acid alleviating copper toxicity in bean seedlings. Proc. Natl. Acad. Sci. India Sec. B Biol. Sci..

[63] Zhang Y., Xu S., Yang S., Chen Y. (2015). Salicylic acid alleviates cadmium-induced inhibition of growth and photosynthesis through upregulating antioxidant defense system in two melon cultivars (*Cucumis melo* L.). Protoplasma.

[64] Horváth E., Pál M., Szalai G., Páldi E., Janda T. (2007). Exogenous 4-hydroxybenzoic acid and salicylic acid modulate the effect of short-term drought and freezing stress on wheat plants. Biol. Plant..

[65] Bartels D., Sunkar R. (2005). Drought and salt tolerance in Plants. CRC Crit. Rev. Plant. Sci..

[66] Tuteja N. (2007). Abscisic acid and abiotic stress signaling. Plant Signal. Behav..

[67] Danquah A., de Zelicourt A., Colcombet J., Hirt H. (2014). The role of ABA and MAPK signaling pathways in plant abiotic stress responses. Biotechnol. Adv..

[68] Rauser W.E., Dumbroff E.B. (1981). Effects of excess cobalt, nickel and zinc on the water relations of *Phaseolus vulgaris*. Environ. Exp. Bot..

[69] Poschenrieder C., Gunsé B., Barceló J. (1989). Influence of cadmium on water relations, stomatal resistance, and abscisic acid content in expanding bean leaves. Plant Physiol..

[70] Hollenbach B., Schreiber L., Hartung W., Dietz K.J. (1997). Cadmium leads to stimulated expression of the lipid transfer protein genes in barley: Implications for the involvement of lipid transfer proteins in wax assembly. Planta.

[71] Stroiński A., Giżewska K., Zielezińska M. (2013). Abscisic acid is required in transduction of cadmium signal to potato roots. Biol. Plant..

[72] Fediuc E., Lips H., Erdei L. (2005). *O*-acetylserine (thiol) lyase activity in Pragmites and Typha plants under cadmium and NaCl stress conditions and the involvement of ABA in the stress response. J. Plant. Physiol..

[73] Stroiński A., Chadzinikolau T., Giżewska K., Zielezińska M. (2010). ABA or cadmium induced phytochelatin synthesis in potato tubers. Biol. Plant..

[74] Kim Y.-H., Khan A.L., Kim D.-H., Lee S.-Y., Kim K.-M., Waqas M., Jung H.-Y., Shin J.-H., Kim J.-G., Lee I.-J. (2014). Silicon mitigates heavy metal stress by regulating P-type heavy metal ATPases, *Oryza sativa* low silicon genes, and endogenous phytohormones. BMC Plant. Biol..

[75] Munzuro Ö., Fikriye K.Z., Yahyagil Z. (2008). The abscisic acid levels of wheat (*Triticum aestivum* L. cv. Çakmak 79) seeds that were germinated under heavy metal (Hg^++^, Cd^++^, Cu^++^) stress. J. Sci..

[76] Wang Y., Wang Y., Kai W., Zhao B., Chen P., Sun L., Ji K., Li Q., Dai S., Sun Y. (2014). Transcriptional regulation of abscisic acid signal core components during cucumber seed germination and under Cu^2+^, Zn^2+^, NaCl and simulated acid rain stresses. Plant Physiol. Biochem..

[77] Atici Ö., Ağar G., Battal P. (2005). Changes in phytohormone contents in chickpea seeds germinating under lead or zinc stress. Biol. Plant..

[78] Monni S., Uhlig C., Hansen E., Magel E. (2001). Ecophysiological responses of *Empetrum nigrum* to heavy metal pollution. Environ. Pollut..

[79] Jeong S., Goto-Yamamoto N., Kobayashi S., Esaka M. (2004). Effects of plant hormones and shading on the accumulation of anthocyanins and the expression of anthocyanin biosynthetic genes in grape berry skins. Plant Sci..

[80] Wheeler S., Loveys B., Ford C., Davies C. (2009). The relationship between the expression of abscisic acid biosynthesis genes, accumulation of abscisic acid and the promotion of *Vitis vinifera* L. berry ripening by abscisic acid. Aust. J. Grape Wine Res..

[81] Giribaldi M., Geny L., Delrot S., Schubert A. (2010). Proteomic analysis of the effects of ABA treatments on ripening *Vitis vinifera* berries. J. Exp. Bot..

[82] Koyama K., Sadamatsu K., Goto-Yamamoto N. (2010). Abscisic acid stimulated ripening and gene expression in berry skins of the Cabernet Sauvignon grape. Funct. Integr. Genom..

[83] Cantín C.M., Fidelibus M.W., Crisosto C.H. (2007). Application of abscisic acid (ABA) at veraison advanced red color development and maintained postharvest quality of ‘Crimson Seedless’ grapes. Postharvest Biol. Technol..

[84] Woźniak A., Formela M., Bilman P., Grześkiewicz K., Bednarski W., Marczak Ł., Narożna D., Dancewicz K., Mai V.Ch., Borowiak-Sobkowiak B. (2017). The dynamics of the defense strategy of pea induced by exogenous nitric oxide in response to aphid infestation. Int. J. Mol. Sci..

[85] Sytykiewicz H. (2008). Influence of bird cherry-oat aphid (Rhaphalosiphum padi (Linnaeus, 1758))/Hemiptera, Aphidoidea/feeding on the activity of β-glucosidase within tissues of its primary host. Aphids Hem. Insects.

[86] Harborne J.B., Cody V., Middleton E., Harborne J. (1978). Nature, distribution and function of plant flavonoids. Plant Flavonoids in Biology and Medicine: Biochemical, Pharmacological, and Structure-Activity Relationships. Progress in Clinical and Biological Research.

[87] Jeandet P. (2015). Phytoalexins: Current progress and future prospects. Molecules.

[88] Jeandet P., Clément C., Courut E., Cordelier S. (2013). Modulation of phytoalexin biosynthesis in engineered plants for disease resistance. Int. J. Mol. Sci..

[89] Adrian M., Jeandet P., Douillet-Breuil A.C., Tesson L., Bessis R. (2000). Stilbene content of mature *Vitis vinifera* berries in response to UV-C elicitation. J. Agric. Food Chem..

[90] Gangwar S., Singh V.P., Prasad S.M., Maurya J.N. (2010). Modulation of manganese toxicity in *Pisum sativum* L. seedlings by kinetin. Sci Hortic..

[91] Zhang Y., Zheng G.H., Liu P., Song J.M., Xu G.D., Cai M.Z. (2011). Morphological and physiological responses of root tip cells to Fe^2+^ toxicity in rice. Acta Physiol Plant..

[92] Gautam S., Anjani K., Srivastava N. (2016). In vitro evaluation of excess copper affecting seedlings and their biochemical characteristics in *Carthamus tinctorius* L. (variety PBNS−12). Physiol. Mol. Biol. Plants..

[93] Ivanov Y.V., Kartashov A.V., Ivanova A.I., Savochkin Y.V., Kuznetsov V.V. (2016). Effects of zinc on Scots pine (*Pinus sylvestris* L.) seedlings grown in hydroculture. Plant Physiol. Biochem..

[94] Mathur S., Kalaji H.M., Jajoo A. (2016). Investigation of deleterious effects of chromium phytotoxicity and photosynthesis in wheat plant. Photosynthetica.

[95] Gwóźdź E.A., Przymusiński R., Rucińska R., Deckert J. (1997). Plant responses to heavy metals: Molecular and physiological aspects. Acta Physiol. Plant..

[96] Rucińska R., Waplak S., Gwóźdź E. (1999). Free radical formation and activity of antioxidant enzymes in lupin roots exposed to lead. Plant. Physiol. Biochem..

[97] Małecka A., Piechalak A., Tomaszewska B. (2009). Reactive oxygen species production and antioxidative defense system in pea root tissues treated with lead ions: The whole roots level. Acta Physiol. Plant..

[98] Shahid M., Pinelli E., Dumat C. (2012). Review of Pb availability and toxicity to plants in relations with metal speciation; role of synthetic and natural organic ligands. J. Hazard. Mater..

[99] Hussain A., Abbas N., Arshad F., Akram M., Khan Z.I., Ahmad K., Mansha M., Mirzaei F. (2013). Effects of diverse doses of lead (Pb) on different growth attributes of *Zea mays* L.. Agric. Sci..

[100] Malar S., Vikram S.S., Favas P.J.C., Perumal V. (2014). Lead heavy metal toxicity induced changes on growth and antioxidative enzymes level in water hyacinths [*Eichhornia crassipes* (Mart.)]. Bot. Stud..

[101] Małecka A., Piechalak A., Morkunas I., Tomaszewska B. (2008). Accumulation of lead in root cells of *Pisum sativum*. Acta Physiol. Plant..

[102] Krzesłowska M., Rabęda I., Basińska A., Lewandowski M., Mellerowicz E.J., Napieralska A., Samardakiewicz S., Woźny A. (2016). Pectinous cell wall thickenings fotmation—A common defense of plants to cope with Pb. Environ. Pollut..

[103] Rabęda I., Bilski H., Mellerowicz E.J., Napieralska A., Suski Sz., Woźny A., Krzesłowska M. (2015). Colocalization of low-methylesterified pectins and Pb deposits in the apoplast of Aspen roots exposed to lead. Environ. Pollut..

[104] Erofeeva E.A. (2014). Hormesis and paradoxical effects of wheat seedling (*Triticum Aestivum* L.) Parameters upon exposure to different pollutants in a wide range of doses. Dose-Response.

[105] Jia L., Liu Z., Chen W., Ye Y., Yu S., He H. (2015). Hormesis effects induced by cadmium on growth and photosyntheticperformance in hypperaccumulator, *Jonicera japonica* Thunb. J. Plant Growth Regul..

[106] Görür G. (2006). Effects of heavy metals accumulation in host plants to cabbage aphid (*Brevicoryne brassicae*)—morphology. Ekologia (Bratislava).

[107] Görür G. (2006). Developmental instability in cabbage aphid (*Brevicoryne brassicae*) populations exposed to heavy metal accumulated host plants. Ecol. Indic..

[108] Mai V.C., Bednarski W., Borowiak-Sobkowiak B., Wilkaniec B., Samardakiewicz S., Morkunas I. (2013). Oxidative stress in pea seedling leaves in response to *Acyrthosiphon pisum* infestation. Phytochemistry.

[109] Yalpani N., León J., Lawton M.A., Raskin I. (1993). Pathway of salicylic acid biosynthesis in healthy and virus-inoculated tobacco. Plant Physiol..

[110] Vine J.H., Niton D., Plummer J.A., Baleriola-Lucas C., Mullins M.G. (1987). Simultaneous quantitation of indole-3-acetic acid and abscisic acid in small samples of plant tissue by gas chromatography/mass spectrometry/selected ion monitoring. Plant Physiol..

[111] Morkunas I., Formela M., Floryszak-Wieczorek J., Marczak Ł., Narożna D., Nowak W., Bednarski W. (2013). Cross-talk interactions of exogenous nitric oxide and sucrose modulates phenylpropanoid metabolism in yellow lupine embryo axes infected with *Fusarium oxysporum*. Plant Sci..

[112] Stobiecki M., Wojtaszek P., Gulewicz K. (1997). Application of solid phase extraction for profiling of quinolisidine alkaloids and phenolic compounds in *Lupinus albus*. Phytochem. Anal..

[113] Morkunas I., Stobiecki M., Marczak Ł., Stachowiak J., Narożna D., Remlein-Starosta D. (2010). Changes in carbohydrate and isoflavonoid metabolism in yellow lupine in response to infection by *Fusarium oxysporum* during the stages of seed germination and early seedling growth. Physiol. Mol. Plant Pathol..

[114] Nichols E.J., Beckman J.M., Hadwiger L.A. (1980). Glycosidic enzyme activity in pea tissue and pea-*Fusarium solani* interactions. Plant Physiol..

[115] Morkunas I., Kozłowska M., Ratajczak L., Marczak Ł. (2007). Role of sucrose in the development of Fusarium wilt in lupine embryo axes. Physiol. Mol. Plant Pathol..

[116] Cahill D.M., McComb J.A. (1992). A comparison of changes in phenylalanine ammonia-lyase activity, lignin and phenolic synthesis in the roots of *Eucalyptus calophylla* (field resistant) and *E. marginata* (susceptible) when infected with *Phytophtora cinnamon*. Physiol. Mol. Plant Pathol..

