# Supplementary material for: The Influence of Lead on Generation of Signalling Molecules and Accumulation of Flavonoids in Pea Seedlings in Response to Pea Aphid Infestation
Source: Molecules. 2017 Aug 24;22(9):1404. doi: 10.3390/molecules22091404 (PMC6151543; doi:10.3390/molecules22091404)
Supplement: Supplementary file 1 [file molecules-22-01404-s001.pdf]

Table S1a Comparisons between particular levels of analysed factor in the leaves of pea seedlings in different times (independently) using the two-sample *t*-test for equal means for all observed traits. Comparisons related to the following plant material variants, i.e. the control vs. the 0.075 mM Pb^{2+} variant; the control vs. 0.5 mM Pb^{2+} variant; the control vs. the +aphids variant; the control vs. 0.075 mM Pb^{2+}+aphids variant; the control vs. 0.5 mM Pb^{2+}+aphids variant;. 0.075 mM Pb^{2+} variant vs. 0.5 mM Pb^{2+} variant; 0.075 mM Pb^{2+} variant vs. 0.075 mM Pb^{2+}+aphids variant; 0.5 mM Pb^{2+} variant vs. 0.5 mM Pb^{2+}+aphids variant; 0.075 mM Pb^{2+}+aphids variant vs. 0.5 mM Pb^{2+}+aphids variant; +aphids variant vs. 0.075 mM Pb^{2+}+aphids variant; +aphids variant vs. 0.5 mM Pb^{2+}+aphids variant

Contrast variant-variant	TSA	SAG	SA	ABA	Pisatin	2'OH-genistein hexoside	Glc-Glc-Glc rhamnose	Glc-Glc-Glc rhamnose iso 1	Glc-Glc-Glc rhamnose iso 2	2'OH-genistein tetrahexosi	Quercetin hexoside	Glc-Glc-Glc kaempferol	Glc-Glc-Glc-Rha quercetin	CHS expression level	PAL expression level	β-glucosidase	PAL	Length	Lead content
Control - 0,075 mM Pb^{2+} (0 h)	-161.8***	-118.5***	-43.2***	15.03***	-0.0165***	0.89	0.8	5.54***	-3.09**	-9.16***	-4.45***	-39.32***	-57.43***	0.01	0.103***	-5.82***	0.566***	-2.73**	0
Control - 0,5 mM Pb^{2+} (0 h)	-322.9***	-215.8***	-106.3***	13.08***	-0.0250***	-24.05***	-5.36***	-4.31***	-5.74***	-24.45***	-9.92***	-43.33***	-14.01***	-0.32***	-0.17***	-6.10***	-0.017	-2.18**	0
0,075 mM Pb^{2+} - 0,5 mM Pb^{2+} (0 h)	-161.1***	-97.3***	-63.1***	-1.95*	-0.0085***	-24.94***	-6.16***	-9.85***	-2.65**	-15.29***	-5.47***	-4.02***	43.42***	-0.33***	-0.273***	-0.27***	-0.583***	0.54	0
Control - +aphids (24 h)	-131.2***	-66.6***	-64.5***	0.9	0.0105***	-0.21	9.01***	4.44***	3.24***	12.83***	0.34	9.25***	7.64***	-0.06**	-0.553***	1.66***	0.08	0.35	-4.2
Control - 0,075 mM Pb^{2+} (24 h)	-26.5***	24.9***	-51.4***	-50.78***	-0.0753***	-86.14***	-5.03***	-22.47***	-9.64***	-70.14***	-7.01***	-34.42***	-6.05***	0.007	-0.457***	5.52***	-0.086	3.87***	-21.5***
Control - 0,075 mM Pb^{2+} +aphids (24 h)	-250.9***	-178.2***	-72.7***	-32.21***	-0.1520***	-52.34***	-1.99*	-8.27***	-6.14***	-38.81***	2.6**	14.01***	-4.27***	-0.06**	-0.273***	4.24***	-0.433***	4.32***	-26.7***
Control - 0,5 mM Pb^{2+} (24 h)	-26.0***	-31.5***	5.9***	1.05	-0.0076**	-21.46***	-0.1	-0.69	4.62***	-3.96***	-6.54***	-25.99***	-1.09	0.703***	0.17***	-2.73***	-0.169	-0.45	2.6
Control - 0,5 mM Pb^{2+}+aphids (24 h)	-78.9***	-52.1***	-26.5***	-9.97***	0.0510***	-9.99***	1.63	4.58***	2.47**	4.91***	-3.26***	-24***	3.95***	0.593***	0.003	-4.83***	-0.609***	-0.21	2.6
+ aphids - 0,075 mM Pb^{2+} +aphids (24 h)	-119.7***	-111.6***	-8.1***	-33.12***	-0.1625***	-52.13***	-11***	-12.71***	-9.38***	-51.64***	2.26*	4.76***	-11.92***	0	0.28***	2.57***	-0.513***	3.97***	-22.5***
+ aphids - 0,5 mM Pb^{2+} +aphids (24 h)	52.3***	14.5***	38.0***	-10.87***	0.0405***	-9.78***	-7.38***	0.13	-0.77	-7.92***	-3.6***	-33.25***	-3.7***	0.653***	0.557***	-6.49***	-0.689***	-0.56	6.9*
0,075 mM Pb^{2+} - 0,075 mM Pb^{2+} +aphids (24 h)	-224.4***	-203.1***	-21.3***	18.57***	-0.0767***	33.8***	3.04**	14.2***	3.51***	31.33***	9.6***	48.43***	1.78*	-0.067**	0.183***	-1.29***	-0.348***	0.46	-5.2*
0,5 mM Pb^{2+} - 0,5 mM Pb^{2+} +aphids (24 h)	-52.9***	-20.6***	-32.4***	-11.01***	0.0586***	11.48***	1.73	5.27***	-2.16*	8.87***	3.28***	1.99***	5.04***	-0.11***	-0.167***	-2.10***	-0.44***	0.24	0
0,075 mM Pb^{2+} - 0,5 mM Pb^{2+} (24 h)	0.5	-56.4***	57.2***	51.83***	0.0677***	64.68***	4.94***	21.79***	14.27***	66.18***	0.46	8.42***	4.96***	0.697***	0.627***	-8.25***	-0.084	-4.31***	24.1***
0,075 mM Pb^{2+} +aphids - 0,5 mM Pb^{2+} +aphids (24 h)	172.0***	126.1***	46.2***	22.25***	0.2029***	42.35***	3.62***	12.85***	8.6***	43.72***	-5.86***	-38.01***	8.22***	0.653***	0.277***	-9.06***	-0.176	-4.53***	29.3***
Control - +aphids (48 h)	-133.8***	-64.8***	-78.9***	1.67	-0.0471***	-90.57***	-6.7***	-2.8***	-1.8*	-7.34***	-3.8***	-6.81***	-7.96***	-0.053*	-0.17***	-0.77***	0.038	0.08	-8.4**
Control - 0,075 mM Pb^{2+} (48 h)	8.4***	-62.5***	70.8***	-14.88***	0.0346***	-65.68***	15.28***	-2.44***	-5.13***	-5.45***	0.13	23.55***	13.91***	0.44***	-0.157***	5.28***	-1.246***	3.28***	3.1
Control - 0,075 mM Pb^{2+} +aphids (48 h)	9.3***	-44.7***	53.7***	-69.81***	-0.0959***	-165.85***	-16.71***	-26.27***	-29.01***	-69.79***	-9.52***	-0.39	-13.96***	-0.02	-0.77***	3.90***	-0.394***	4.18***	-32.5***
Control - 0,5 mM Pb^{2+} (48 h)	-683.9***	-478.7***	-205.0***	-83.5***	-0.1283***	-121.05***	-11.87***	-13.59***	-11.81***	-44.05***	3.31***	26.27***	-20.12***	-0.087***	-0.48***	4.84***	-1.866***	5.05***	-62.5***
Control - 0,5 mM Pb^{2+} +aphids (48 h)	-14.8***	-14.6***	0.2	-33.1***	-0.0434***	-106.73***	7.6***	-18.26***	-17.67***	-40.98***	-7.17***	23.03***	5.16***	0.537***	-0.35***	5.63***	-1.964***	4.94***	-14.2***
+ aphids - 0,075 mM Pb^{2+} +aphids (48 h)	143.0***	20.2***	132.5***	-71.48***	-0.0488***	-75.28***	-10.01***	-23.47***	-27.21***	-62.45***	-5.72***	6.41***	-6***	0.033	-0.6***	4.67***	-0.432***	4.1***	-24.2***
+ aphids - 0,5 mM Pb^{2+} +aphids (48 h)	119.0***	50.3***	79.1***	-34.77***	0.0037	-16.17***	14.3***	-15.45***	-15.87***	-33.64***	-3.37***	29.83***	13.12***	0.59***	-0.18***	6.40***	-2.002***	4.86***	-5.8*
0,075 mM Pb^{2+} - 0,075 mM Pb^{2+} +aphids (48 h)	0.9	17.8***	-17.2***	-54.93***	-0.1305***	-100.17***	-31.99***	-23.83***	-23.88***	-64.35***	-9.65***	-23.94***	-27.86***	-0.46***	-0.613***	-1.39***	0.852***	0.9	-35.6***
0,5 mM Pb^{2+} - 0,5 mM Pb^{2+} +aphids (48 h)	669.1***	464.2***	205.2***	50.4***	0.0849***	14.31***	19.48***	-4.67***	-5.86***	3.07**	-10.48***	-3.24**	25.28***	0.623***	0.13***	0.79***	-0.098	-0.11	48.3***
0,075 mM Pb2+ - 0,5 mM Pb^{2+} (48 h)	-692.3***	-416.2***	-275.8***	-68.62***	-0.1630***	-55.37***	-27.15***	-11.15***	-6.68***	-38.61***	3.18**	2.72**	-34.03***	-0.527***	-0.323***	-0.45***	-0.62***	1.77*	-65.6***
0,075 mM Pb^{2+} +aphids - 0,5 mM Pb^{2+} +aphids (48 h)	-24.0***	30.1***	-53.4***	36.71***	0.0525***	59.12***	24.31***	8.01***	11.34***	28.81***	2.35*	23.42***	19.12***	0.557***	0.42***	1.73***	-1.57***	0.76	18.3***
Control - +aphids (72 h)	-87.2***	-29.8***	-57.6***	-2.76**	0.0031	-31.01***	-5.32***	-11.55***	-6.04***	-23.79***	-10.31***	-10.11***	-7.04***	-0.27***	-0.24***	-3.47***	-0.469***	0.23	0
Control - 0,075 mM Pb^{2+} (72 h)	22.7***	72.2***	-50.1***	-3.12***	0.0185***	-35.35***	-7.22***	-11.64***	-7.23***	-20.11***	-12.76***	-21.05***	-6.11***	-0.7***	-0.257***	0.34***	-0.31**	-0.65	-1.8
Control - 0,075 mM Pb^{2+} +aphids (72 h)	-44.6***	-3.9	-40.6***	-8.04***	0.0246***	-33.86***	-12.74***	-16.05***	-1.84*	-23.93***	-7.65***	-11.08***	-13.31***	-0.83***	-0.23***	-1.03***	-0.549***	-0.15	-4.6
Control - 0,5 mM Pb^{2+} (72 h)	-9.0***	53.2***	-62.4***	-93.01***	-0.0396***	-90.13***	-15.27***	-30***	-18.08***	-73.87***	1.08	-4.7***	-18.34***	-0.573***	-0.567***	2.81***	-0.392***	2.88***	-29.7***
Control - 0,5 mM Pb^{2+} +aphids (72 h)	-211.6***	-92.8***	-118.4***	-13.78***	-0.0758***	-74.69***	-15.94***	-22.46***	-13.97***	-64.12***	-10.72***	-6.54***	-19.45***	-0.843***	-0.21	3.43***	-1.605***	3.7***	-38.1***
+ aphids - 0,075 mM Pb^{2+} +aphids (72 h)	42.6***	25.8***	17.0***	-5.28***	0.0215***	-2.86**	-7.42***	-4.5***	4.21***	-0.14	2.66**	-0.96	-6.28***	-0.56***	0.01	2.44***	-0.08	-0.38	-4.6
+ aphids - 0,5 mM Pb^{2+} +aphids (72 h)	-124.4***	-63.1***	-60.8***	-11.01***	-0.0789***	-43.68***	-10.62***	-10.91***	-7.93***	-40.33***	-0.41	3.57***	-12.41***	-0.573***	0.03	6.90***	-1.136***	3.47***	-38.1***
0,075 mM Pb^{2+} - 0,075 mM Pb^{2+} +aphids (72 h)	-67.3***	-76.1***	9.5***	-4.92***	0.0061**	1.49	-5.51***	-4.41***	5.39***	-3.82***	5.1***	9.97***	-7.2***	-0.13***	0.027	-1.37***	-0.239*	0.5	-2.8
0,5 mM Pb^{2+} - 0,5 mM Pb^{2+} +aphids (72 h)	-202.5***	-146***	-56.0***	79.23***	-0.0362***	15.45***	-0.67	7.54***	4.11***	9.75***	-11.8***	-1.84	-1.11	-0.27***	0.357***	0.62***	-1.213***	0.82	-8.4**
0,075 mM Pb^{2+} - 0,5 mM Pb^{2+} (72 h)	-31.7***	-19***	-12.4***	-89.89***	-0.0582***	-54.78***	-8.04***	-18.36***	-10.85***	-53.75***	13.84***	16.34***	-12.23***	0.127***	-0.31***	2.47***	-0.082	3.52***	-28***
0,075 mM Pb^{2+} +aphids - 0,5 mM Pb^{2+} +aphids (72 h)	-166.9***	-88.9***	-77.8***	-5.74***	-0.1004***	-40.83***	-3.2***	-6.41***	-12.13***	-40.19***	-3.06**	4.54***	-6.14***	-0.013	0.02	4.46***	-1.056***	3.85***	-33.5***

*** P<0.05; ** P<0.01; *** P<0.001**

Table S1b Comparisons between particular levels of analysed factors in the roots of pea seedlings in different times (independently) using the two-sample *t*-test for equal means for all observed traits. Comparisons related to the following plant material variants, i.e. the control vs. the 0.075 mM Pb^{2+} variant; the control vs. 0.5 mM Pb^{2+} variant; the control vs. the +aphids variant; the control vs. 0.075 mM Pb^{2+}+aphids variant; the control vs. 0.5 mM Pb^{2+}+aphids variant;. 0.075 mM Pb^{2+} variant vs. 0.5 mM Pb^{2+} variant; 0.075 mM Pb^{2+} variant vs. 0.075 mM Pb^{2+}+aphids variant; 0.5 mM Pb^{2+} variant vs. 0.5 mM Pb^{2+}+aphids variant; 0.075 mM Pb^{2+}+aphids variant vs. 0.5 mM Pb2++aphids variant; +aphids variant vs. 0.075 mM Pb2++aphids variant; +aphids variant vs. 0.5 mM Pb^{2+}+aphids variant

Contrast variant-variant	TSA	SAG	SA	ABA	Pisatin	2'OH-genistein hexoside	Glc-Glc-Glc rhamnose	Glc-Glc-Glc rhamnose iso 1	2'OH-genistein tetrahexoside	quercetin hexoside	Glc-Glc-Glc kaempferol	Glc-Glc-Glc-Rha quercetin	β-glucosidase	PAL	Length	Lead content
Control - 0,075 mM Pb^{2+} (0 h)	-13	-7.29***	-8.6***	0	-0.159*	4.38***	3.259***	1.795***	1.35***	0.086***	4.27***	1.525***	1.16***	51.6***	-2.1	0
Control - 0,5 mM Pb^{2+} (0 h)	-85	-81.93***	-4.4*	0	-0.131	4.04***	3.423***	1.906***	1.31***	0.083***	3.14***	1.547***	2.06***	56.7***	-1.2	0
0,075 mM Pb^{2+} - 0,5 mM Pb^{2+} (0 h)	-72	-74.64***	4.2*	0	0.028	-0.34**	0.164***	0.111	-0.04*	-0.002*	-1.13***	0.022	0.89***	5.1*	0.9	0
Control - +aphids (24 h)	205	194.25***	11***	0	0.236**	-0.04	-0.065*	-0.115	-0.01	0.004***	3.16***	-0.002	-0.96***	-10.3***	-0.3	657.1***
Control - 0,075 mM Pb^{2+} (24 h)	-9361***	-7221.21***	-2139.7***	0	-4.474***	-3.8***	-0.917***	-0.895***	-0.31***	0.000	7.04***	-0.224***	-23.01***	35.2***	5.9**	-19322***
Control - 0,075 mM Pb^{2+} +aphids (24 h)	-7541***	-7045.34***	-495.7***	-2.95***	-3.086***	-1.53***	-0.181***	-0.09	-0.15***	0.004***	7.41***	-0.139**	-22.26***	49.4***	4.7**	-17976***
Control - 0,5 mM Pb^{2+} (24 h)	211	200.1***	11.4***	-1.66***	0.253**	-1.35***	-0.082**	-0.015	-0.20***	0.004***	4.93***	-0.07	-1.08***	-34***	-2.1	5589.7***
Control - 0,5 mM Pb^{2+} +aphids (24 h)	175	162.51***	12.8***	-1.94***	0.383***	-1.36***	0.068*	0.029	-0.13***	0.002	6.79***	-0.009	-2.05***	-19.7***	-1.6	5589.7***
+ aphids - 0,075 mM Pb^{2+} +ahids (24 h)	-7746***	-7239.59***	-506.7***	-2.95***	-3.322***	-1.49***	-0.115***	0.025	-0.14***	0.000	4.25***	-0.137**	-21.29***	59.8***	5**	-18633.1***
+ aphids - 0,5 mM Pb^{2+} +aphids (24 h)	-30	-31.74***	1.8	-1.94***	0.147	-1.33***	0.133***	0.144	-0.12***	-0.003*	3.63***	-0.008	-1.09***	-9.4***	-1.3	4932.5***
0,075 mM Pb^{2+} - 0,075 mM Pb^{2+} +aphids (24 h)	1820*	175.87***	1644***	-2.95***	1.387***	2.27***	0.737***	0.805***	0.16***	0.004***	0.37*	0.085*	0.76***	14.3***	-1.2	1346.1***
0,5 mM Pb^{2+} - 0,5 mM Pb^{2+} +aphids (24 h)	-37	-37.59***	1.4	-0.28*	0.13	-0.01	0.15***	0.044	0.07**	-0.003*	1.86***	0.06	-0.97***	14.3***	0.5	0
0,075 mM Pb^{2+} - 0,5 mM Pb^{2+} (24 h)	9572***	7421.31***	2151.1***	-1.66***	4.726***	2.45***	0.835***	0.88***	0.11***	0.004***	-2.11***	0.154***	21.93***	-69.2***	-7.9***	24911.7***
0,075 mM Pb^{2+} +aphids - 0,5 mM Pb^{2+} +aphids (24 h)	7716***	7207.85***	508.5***	1.01***	3.469***	0.17	0.249***	0.119	0.02	-0.003*	-0.62***	0.13**	20.21***	-69.2***	-6.2***	23565.6***
Control - +aphids (48 h)	4	10.04***	-6.2**	1.21***	0.222**	-0.54***	-0.26***	-0.296**	-0.21***	0.000	-2.99***	-0.107*	-0.02	42***	0.9	621.7***
Control - 0,075 mM Pb^{2+} (48 h)	-9	-4.13***	-4.4*	3.85***	0.158*	-1.09***	-0.345***	-0.066	-0.04	-0.003*	2.07***	-0.026	-3.20***	-2.7	3	176.4***
Control - 0,075 mM Pb^{2+} +aphids (48 h)	-5767***	-8059.57***	-560.9***	1.5***	-4.464***	-2.51***	-0.443***	-0.561***	-0.22***	-0.003**	0.96***	-0.051	-28.71***	94.1***	7.3***	-22546.5***
Control - 0,5 mM Pb^{2+} (48 h)	-10529***	-10000.09***	-530.1***	3.85***	-2.741***	-2.33***	-0.644***	-0.377***	-0.11***	-0.004**	2.11***	-0.06	-21.94***	92.9***	7.4***	-19972.6***
Control - 0,5 mM Pb^{2+} +aphids (48 h)	-8285***	-5709.86***	-2577.4***	2.38***	-1.484***	-2.37***	-0.719***	-0.665***	-0.14***	0.000	2.51***	-0.15***5	-7.36***	80.9***	7.4***	-13363.8***
+ aphids - 0,075 mM Pb^{2+} +aphids (48 h)	-5772***	-8069.61***	-554.7***	0.29*	-4.687***	-1.98***	-0.184***	-0.266**	-0.01	-0.003**	3.94***	0.056	-28.69***	52***	6.4***	-23168.2***
+ aphids - 0,5 mM Pb^{2+} +aphids (48 h)	-8290***	-5719.9***	-2571.2***	1.17***	-1.707***	-1.83***	-0.46***	-0.369***	0.07**	0.000	5.5***	-0.048	-7.34***	38.8***	6.4***	-13985.5***
0,075 mM Pb^{2+} - 0,075 mM Pb^{2+}+aphids (48 h)	-5758***	-8055.44***	-556.5***	-2.35***	-4.623***	-1.42***	-0.098**	-0.495***	-0.19***	-0.001	-1.11***	-0.025	-25.51***	96.8***	4.3*	-22722.9***
0,5 mM Pb^{2+} - 0,5 mM Pb^{2+} +aphids (48 h)	2244*	4290.23***	-2047.3***	-1.47***	1.257***	-0.05	-0.075*	-0.288**	-0.03	0.004**	0.39*	-0.095*	14.58***	-12.1***	-0.1	6608.8***
0,075 mM Pb^{2+} - 0,5 mM Pb^{2+} (48 h)	-10520***	-9995.96***	-525.7***	0	-2.899***	-1.23***	-0.299***	-0.311***	-0.07**	-0.001	0.04	-0.034	-18.74***	95.7***	4.4*	-20149***
0,075 mM Pb^{2+} +aphids - 0,5 mM Pb^{2+} +aphids (48 h)	-2518**	2349.71***	-2016.6***	0.88***	2.98***	0.14	-0.276***	-0.104	0.09***	0.003**	1.55***	-0.104*	21.35***	-13.2***	0.1	9182.7***
Control - +aphids (72 h)	96	91.3***	3.9	-1.9***	-0.057	-0.65***	-0.262***	-0.298**	0.06**	0.000	-0.69***	-0.007	0.38***	5.1*	0.8	0
Control - 0,075 mM Pb^{2+} (72 h)	93	86.11***	6.3**	0	-0.125	0.17	-0.033	-0.12	-0.02	0.000	-3.21***	-0.011	-0.02	27.1***	0.7	-4344.5***
Control - 0,075 mM Pb^{2+} +aphids (72 h)	-124	-87.84***	-36.1***	0	-0.133	-0.81***	-0.06*	-0.05	-0.02	-0.004**	-1.68***	-0.146***	0.30***	37***	0.4	-4901.8***
Control - 0,5 mM Pb^{2+} (72 h)	-9379***	-6330.83***	-3048.2***	-14.82***	-3.28***	-2.24***	-0.537***	-0.784***	-0.30***	-0.006***	-0.91***	-0.259***	-13.06***	47***	3.5*	-24573.2***
Control - 0,5 mM Pb^{2+} +aphids (72 h)	-6858***	-5681.2***	-1177.1***	-2.68***	-2.298***	-2.27***	-0.456***	-0.546***	-0.24***	-0.004***	1***	-0.228***	-20.27***	89***	5.5**	-25748.2***
+ aphids - 0,075 mM Pb^{2+} +aphids (72 h)	-221	-179.14***	-40***	1.9***	-0.077	-0.16	0.201***	0.248**	-0.08***	-0.004**	-0.98***	-0.139**	-0.08	31.9***	-0.4	-4901.8***
+ aphids - 0,5 mM Pb^{2+} +aphids (72 h)	-6955***	-5772.5***	-1181***	-0.78***	-2.242***	-1.62***	-0.194***	-0.248**	-0.30***	-0.004***	1.69***	-0.221***	-20.65***	83.9***	4.7**	-25748.2***
0,075 mM Pb^{2+} - 0,075 mM Pb^{2+} +aphids (72 h)	-217	-173.95***	-42.5***	0	-0.008	-0.98***	-0.027	0.07	0.00	-0.004**	1.53***	-0.136**	0.32***	9.8***	-0.3	-557.3***
0,5 mM Pb^{2+} - 0,5 mM Pb^{2+} +aphids (72 h)	2520**	649.63***	1871***	12.14***	0.981***	-0.03	0.081**	0.238**	0.06*	0.002	1.91***	0.031	-7.21***	42***	2	-1175***
0,075 mM Pb^{2+} - 0,5 mM Pb^{2+} (72 h)	-9471***	-6416.94***	-3054.5***	-14.82***	-3.154***	-2.41***	-0.504***	-0.664***	-0.27***	-0.006***	2.3***	-0.248***	-13.04***	19.9***	2.8	-20228.7***
0,075 mM Pb^{2+} +aphids - 0,5 mM Pb^{2+} +aphids (72 h)	-6734***	-5593.36***	-1141***	-2.68***	-2.165***	-1.46***	-0.396***	-0.496***	-0.22***	-0.001	2.67***	-0.082*	-20.57***	52***	5.1**	-20846.3***

*** P<0.05; ** P<0.01; *** P<0.001**

Table S2 Comparison between variant combinations on the basis of lead content in bodies of aphids. Comparisons related to the following aphid variants, i.e., the control vs. 0.075 mM Pb^{2+} variant; the control vs. 0.5 mM Pb^{2+} variant; 0.075 mM Pb^{2+} variant vs. 0.5 mM Pb^{2+} variant, respectively

**Contrasts variant-variant**	**Lead content**
control - 0,075 mM Pb^{2+} (24 h)	-23
control - 0,5 mM Pb^{2+} (24 h)	-174***
0,075 mM Pb^{2+} - 0,5 mM Pb^{2+} (24 h)	-151***
control - 0,075 mM Pb^{2+} (48 h)	-30*
control - 0,5 mM Pb^{2+} (48 h)	-436***
0,075 mM Pb^{2+} vs. 0,5 mM Pb^{2+} (48 h)	-406***
control - 0,075 mM Pb^{2+} (72 h)	-25*
control - 0,5 mM Pb^{2+} (72 h)	-444***
0,075 mM Pb^{2+} - 0,5 mM Pb^{2+} (72 h)	-419***
*** P<0.05; *** P<0.001**	
